# Sanye Tablet Ameliorates Insulin Resistance and Dysregulated Lipid Metabolism in High-Fat Diet-Induced Obese Mice

**DOI:** 10.3389/fphar.2021.713750

**Published:** 2021-09-29

**Authors:** Minghe Yao, Lin Li, Ming Huang, Yao Tan, Ye Shang, Xianghui Meng, Yafen Pang, Hong Xu, Xin Zhao, Wei Lei, Yanxu Chang, Yi Wang, Deqin Zhang, Boli Zhang, Yuhong Li

**Affiliations:** ^1^ State Key Laboratory of Component-based Chinese Medicine, Tianjin University of Traditional Chinese Medicine, Tianjin, China; ^2^ Key Laboratory of Pharmacology of Traditional Chinese Medical Formula, Ministry of Education, Tianjin University of Traditional Chinese Medicine, Tianjin, China

**Keywords:** SYT, HFD, insulin resistance, lipidomics, proteomics

## Abstract

Sanye Tablet (SYT) is a patent prescription widely used in treating T2D and pre-diabetes, especially T2D comorbid with hypertriglyceridemia, for many years in China. However, the underlying mechanism that accounts for the anti-diabetic potential of SYT by regulating lipid-related intermediates remains to be elucidated. This study aimed to investigate the mechanism of SYT on lipid metabolism and insulin sensitivity in high-fat diet (HFD)-induced obese mice by means of combining lipidomics and proteomics. The obese mice models were developed via HFD feeding for 20 consecutive weeks. Mice in the treatment group were given metformin and SYT respectively, and the effects of SYT on body weight, blood glucose, insulin sensitivity, fat accumulation in the organs, and pathological changes in the liver were monitored. Lipid metabolism was examined by lipidomics. Further determination of signaling pathways was detected by proteomics. The biological contributions of the compounds detected in SYT’s chemical fingerprint were predicted by network pharmacology. SYT treatment reduced body weight, inhibited viscera and hepatic steatosis lipid accumulation, and prevented insulin resistance. Furthermore, it was found that circulatory inflammatory cytokines were reduced by SYT treatment. In addition, lipidomics analysis indicated that SYT targets lipid intermediates, including diacylglycerol (DAG) and Ceramide (Cer). Mechanistically, SYT positively affected these lipid intermediates by suppressing liver lipogenesis via downregulation of SREBP1/ACC and the JAK/STAT signaling pathway. Our results predicted that astragalin and rosmarinic acid might regulate the JAK-STAT pathway by targeting PIM2 and STAT1, respectively, while paeoniflorin and rosmarinic acid were likely to regulate inflammatory responses by targeting TNFα, IL-6, and IL-4 during T2D. Overall, our study provides supportive evidence for the mechanism of SYT’s therapeutic effect on dysregulated lipid metabolism in diabesity.

## Introduction

According to the latest epidemiologic data from the International Diabetes Federation, there are 463 million diabetics worldwide. It has gradually become a public health problem that seriously affects the global population and brings great economic burden to society (Saeedi et al., 2019). Type 2 diabetes mellitus (T2D) accounts for more than 90 percent of diabetes. Thus, it is of great significance to study the pathophysiological mechanisms, effective prevention, and treatment strategies of T2D. T2D is characterized by relatively deficient insulin secretion from pancreatic islet β-cells combined with insulin resistance in target organs (Li et al., 2020). Its dominant cause is obesity, with ectopic fat accumulation in vital organs. The formation of obesity is a long process. Diet, exercise, and other factors can lead to the occurrence of obesity. The western diet, especially, featured by high fat and high sugar foods, has been widely considered the contributing factor to the recent rapid increase in obesity (Lean, 2019).

Long-term excess intake of high-calorie or high-fat diet results in increased blood glucose level and fatty acid content. The increased blood glucose level caused continuous insulin secretion from islet cells, while the increased fatty acid and lipid content are the main culprits for IR (You et al., 2021). In the beginning, insulin exerts its hypoglycemic function by binding with its receptors in the liver and skeletal muscle cells in order to promote glucose absorption and utilization. However, the persistent accumulation of fatty acid and lipid content in systemic circulation influences metabolic cells and tissues in numerous ways, notably including acceleration of systematic inflammation and promotion of lipogenesis in the liver, the leading lipid synthesis site. Massive accumulation of lipids in the liver also further aggravates inflammatory responses, resulting in insulin resistance (Chang et al., 2015).

Medical management of diabesity often requires lifetime administration of medications. A wealth of evidence has confirmed that traditional Chinese medicine (TCM) is an effective strategy for ameliorating insulin resistance. The importance of TCM in diabesity is of profound interest because TCM-medicinal products seem to prevent diabesity in a relatively safe manner with long-term benefits. Thus, the development of TCM-medicinal products as anti-diabetic alternatives has been gaining more attention in basic biomedical and clinical research.

A Chinese herbal medicine formula consisting of *Morus alba L.* Leaf, *Nelumbo nucifera Gaertn.* Leaf, *Crataegus pinnatifida Bunge* Leaf, *Salvia miltiorrhiza Bunge* Root, *Paeonia lactiflora Pall.* Root is a widely used TCM prescription in treating T2D and pre-diabetes for many years in China, especially in treating patients with hypertriglyceridemia. This Chinese herbal formula has been refined and processed into a tablet named Sanye Tablet (SYT) or Tangzhiqing Tablet (TZQ) as a new patent traditional Chinese medical formula. An *in vitro* experiment demonstrated that 2,3,4,6-tetra-O-galloyl-D-glucose, 1,2,3,4-tetra-O-galloyl-D-glucose, 1,2,3,4,6-penta-O-galloyl-d-glucose, quercetin-3-O-β-D-glucuronide and quercetin-3-O-β-D-glucoside were the primary biological active components of SYT due to the inhibitory effects on maltase, invertase, and lipase (Tao et al., 2013). After oral administration of SYT, 86 components were identified in rat plasma, urine, and feces, including alkaloids, flavonoids, phenolic acid, diterpenoid quinones, monoterpenoids, and metabolites of glucuronidation and sulfation, which were found to be absorbed components (Wu et al., 2018). Nuciferine and paeoniflorin were suggested as the quality markers of SYT (Li et al., 2018). Our previous *in vitro* pharmacological study has shown that SYT exhibits biological activities on inhibiting intestinal disaccharidase and lipase and scavenging free radicals (Tao et al., 2010).

Moreover, recent insights into the biological importance of SYT on improving hyperglycemia, dyslipidemia, and insulin resistance have been reported and suggest that SYT could be likely regulating the expression of genes related to the insulin signaling pathway in the liver and muscle (Nan et al., 2013; Gao et al., 2014) and lipid metabolism (An et al., 2013). Importantly, our previous clinical research indicated that the clinical indication of SYT was T2D with hypertriglyceridemia and with the speculation that SYT might be regulating glycerophospholipid metabolism (Liu et al., 2018). However, little is known about the biological influence of SYT on lipid metabolism and insulin resistance. Therefore, based on the available preclinical and clinical data, the purpose of the present study was to explore the underlying mechanism of SYT on lipid metabolism by combining lipidomics and proteomics in HFD-induced insulin resistance mice.

## Materials and Methods

### Drugs and Reagents

SYT was provided by Shandong Buchang Pharmaceuticals Co., Ltd (Shandong, China). Metformin (1, 1-Dimethylbiguanide hydrochloride) was purchased from Beijing Solarbio Science & Technology Co. Ltd (Beijing, China). Anhydrous glucose was purchased from Tianjin Damao Chemical Reagent Factory (Tianjin, China). Insulin was purchased from Novo Nordisk (Copenhagen, Denmark). Anhydrous alcohol and xylene were purchased from Tianjin Fengchuan Chemical Reagent Technologies Co. Ltd (Tianjin, China). HPLC-grade methanol, acetonitrile, and isopropanol were obtained from Thermofisher Scientific Co. Ltd (Pittsburgh, PA, United States). MS-grade ammonium acetate was obtained from Sigma-Aldrich Trading Co. Ltd (Shanghai, China). All other analytical grade chemicals were purchased from Sinopharm Chemical Reagent Co. Ltd (Beijing, China). Leptin, insulin, resistin, and interleukin (IL)-1α mouse high sensitivity T cell panel-immunology multiplex assay were purchased from Merck KGaA (Darmstadt, Germany). The Alanine Transaminase (ALT) kit was supplied by Zhongsheng Beikong Bio-technology and Science, Inc. (Beijing, China). The mouse LPS Elisa kit was obtained from Shanghai Huyu Biotechnology Co. Ltd (Shanghai, China). The Hematoxylin and Eosin (HE) and oil red O stain kits were purchased from Beijing Solarbio Science & Technology Co., Ltd (Beijing, China). RIPA lysis buffer (medium), TBST (pH 8.0, 10×), tris-glycine transfer buffer (pH 8.3, 10×), Tris-glycine SDS buffer (pH 8.3, 10×), tricine-SDS-PAGE gel kit, and BCA protein assay kit were purchased from CoWin Biosciences, Inc. (Beijing, China). Sterol regulatory element-binding protein-1 (SREBP-1) antibody (A-4) and β-Actin (c4) were purchased from Santa Cruz Biotechnology, Inc. (California, United States). Acetyl-CoA carboxylase antibody (ACC), anti-rabbit IgG HRP-linked antibody, and anti-mouse IgG HRP-linked antibody were purchased from Cell Signaling Technology, Inc. (Massachusetts, United States). Purified water was obtained from Milli-Q integral system (Darmstadt, Germany).

### Animals and Treatments

Male C57BL/6N mice, aged 5–6 weeks (weighing 18–20 g), were purchased from the Beijing Vital River Laboratory Animal Technology Co., Ltd (Beijing, China). These mice were housed in plastic cages of five mice each under standardized housing conditions with a consistent temperature of 20–26°C, relative humidity of 40–70%, and a 12 h light/dark cycle with free access to food and water. All animal experiments conformed to the ethical requirements and regulations of the Animal Experimental Ethics Committee of Tianjin University of TCM (Animal Experiment Ethics No. TCM-LAEC2020050).

After 1 week of adaptive feeding with a basal diet, mice were randomly divided into five groups: basal diet group (Con), high-fat diet (HFD) group, HFD with 100 mg/kg metformin (MET) group, HFD with 0.2 g/kg SYT (SYTL) group, and HFD with 0.4 g/kg SYT (SYTH) group. Mice in the Con group were given basal diet (13.5% of energy from fat, the heat is 3.0 kcal/gm; purchased from Beijing SPF Biotechnology Co., Ltd), while those in other groups were given HFD (60% of energy from fat, the heat is 5.24 kcal/gm; purchased from Beijing HFK Bioscience Co., Ltd). MET and SYT were prepared in purified water and given once daily by gastric gavage for 20 consecutive weeks. Mice in the Con group and HFD group were given purified water as control. Body weight was recorded once a week, while food was weighed daily. Food intake was calculated by subtracting the amount of residue from the amount of supply food.

### Glucose Tolerance Test (GTT) and Insulin Tolerance Test (ITT)

GTT and ITT were performed at the end of week 20. Mice fasted for 12 h or 6 h and were intraperitoneally injected with glucose at 2 g/kg body mass or human insulin 0.75 U/kg. Blood was obtained from the tail vein at 0, 15, 30, 60, 90, and 120 min after glucose or insulin administration. Blood glucose concentration was determined by a glucose meter (Roche Diagnostics GmbH, Mannheim, Germany). The curve of glucose concentration over time was plotted, and the area under the curve (AUC) was calculated for each animal.

### Biochemical Analysis

At the end of the experiment, the mice were anesthetized with tribromoethanol solution. Blood was collected from the orbital vein of the unconscious mice. Then, serum was separated and stored at −80°C until further analysis of biochemical parameters. Serum insulin, leptin, resistin, and IL-1α were detected using the mouse high sensitivity T cell panel-immunology multiplex assay. Serum ALT was determined by Hitachi Chemistry Analyzer 7060 (Hitachi, Tokyo, Japan). Serum LPS was measured by an Elisa kit. The homeostasis model assessment of insulin resistance (HOMA-IR) index was calculated using the following equation: HOMA-IR = fasting serum glucose (mmol/L) × fasting serum insulin (mU/mL)/22.5 (Neyrinck et al., 2012).

### Viscera Weight and Visceral Index

After blood collection, liver and adipose tissues (epididymal fat and subcutaneous fat) were promptly removed and weighed. The visceral index was calculated as tissues/organs wet weight (g)/body weight (g) × 100%.

### HE and Oil Red O Staining

Liver and epididymal fat were fixed in 10% neutral buffered formalin for 24 h. Fixed tissues were embedded in paraffin, sectioned 4 μm thick, and stained with HE. The fixed tissues were then embedded in OCT, sectioned, and stained with oil red O. All images were observed using a Leica DM 2500 microscope (Leica Microsystems Co., Ltd, Bensheim, Germany).

### Western Blotting

Proteins from the liver were extracted. Equal amounts of protein were subjected to sodium dodecyl sulfate-polyacrylamide gel electrophoresis and electro-transferred to polyvinylidene difluoride membranes. After blocking with 5% skim milk for 2 h, membranes were incubated with primary antibodies at 4°C overnight. Membranes were then washed with TBST repeatedly and incubated with horseradish peroxidase-conjugated secondary antibody. Images were determined by an electrochemiluminescence system.

### UPLC Fingerprint of SYT

#### Sample Preparation

The water-soluble extract of the SYT formula was centrifuged for 10 min at 14,000 rpm, and the supernatant was collected. The supernatant was filtered with a 0.22 μm filter membrane and diluted by 40 times with water. An aliquot (2 μl) of the supernatant solution was injected into UPLC-PDA for analysis.

#### UPLC Analysis

An Ultra Performance Liquid Chromatography (UPLC) system (Waters) consisting of a quaternary pump, autosampler, thermostatted column compartment, and PDA detector was employed throughout the analysis. All separation was performed on an ACQUITYUPLCBEH C18 column. The flow rate was set as: 0.3 ml/min at 0–10 min, 0.3–0.4 ml/min at 10–12 min, and 0.4 ml/min at 12–36 min. Column temperature was 40°C. The mobile phase comprised of (A) aqueous formic acid (0.1%, v/v) and (B) acetonitrile using a gradient elution of 5–8% B at 0–2 min, 8–10% B at 2–5 min, 10–10% B at 5–8 min, 10–11.5% B at 8–10 min, 11.5–11.7% B at 10–12 min, 11.7–12% B at 12–18 min, 12–16% B at 18–20 min, 16–17% B at 20–25 min, 17–18% B at 25–32 min, 18–22% B at 32–33 min, 22–95% B at 33–35 min, and 95–5% B at 35–36 min. The re-equilibration time of gradient elution was 5 min. Moreover, the detection wavelength was set at 270 and 230 nm (Liu et al., 2020).

### Lipidomics Analysis Based on UHPLC-MS

#### Sample Preparation

The serum samples stored at −80°C were thawed in a 1.5 ml centrifuge tube. For the extraction of serum lipids, 300 μl of precooled isopropanol was added, and the resulting solution was vortexed for 5 min and kept at −20°C for 1 h, followed by 20 min of 13,000 rpm centrifugation at 4°C for 20 min. The obtained organic phase (upper isopropanol layer) was transferred into a new vial, and 2 μl was injected into UHPLC-MS for lipid quantification and analysis. Furthermore, 10 μl was pooled and processed for each sample to obtain lipidomics QC samples.

#### UHPLC-MS Instrument Conditions

The liquid chromatogram analysis was performed on a Shimadzu LC-30AD system (Shimadzu, Kyoto, Japan). ACQUITY UPLC BEH C8 column (Waters, 1.7 μm, 2.1 × 100 mm) was used for separation. Mobile phase A consisted of a mixture of water:methanol:acetonitrile (3:1:1, v/v/v) while mobile phase B was isopropanol, with both containing 5 mM ammonium acetate. The mobile phase flow rate was 0.3 ml/min, sample injection volume was 2 μl, and elution conditions were as follows: 0–0.5 min, 20% B; 0.5–1.5 min, 20–40% B; 1.5–3 min, 40–60% B; 3–13 min, 60–100% B; 13–14 min, 100% B; 14–17 min, 20% B.

Mass spectrometry was conducted on a triple quadrupole linear ion trap mass spectrometer (QTRAP 6500+) (AB SCIEX, Framingham, MA, United States) coupled with an ESI source. Scheduled multi-reaction monitoring (MRM) with chosen time windows under either positive or negative ion modes was utilized for acquire for the acquisition of chromatograms. MS parameters were configured with gas temperature at 400°C, ion spray voltage at 5,500 V, and with the ion source gas I (GSI), the gas II (GSII), and the curtain gas (CUR) corresponding to 50, 50, and 35 psi respectively.

#### MS Data Processing

Chromatogram intensities were converted into raw data using the XCMS package in R, and data were subsequently analyzed with Analyst 1.6.2 software. Peak area derivation was evaluated by Sciex OS1.4 software (AB SCIEX, Framingham, MA, United States), and MetaboAnalyst 4.0 statistical package was used for data filtration and sum normalization. The refined data were further analyzed using relative quantification methods, and multivariate statistical analysis performed was identical to the procedure mentioned earlier using SIMCA.

### TMT-Labeled Quantitative Proteomics

#### Protein Extraction

Three samples of liver tissue in each group were selected for proteomics detection. Protein was extracted by SDT (4% SDS, 150 mM Tris/HCl pH 8.0, 1 mM DTT, and protease inhibitor) lysis method. Protein content was determined with the BCA protein assay reagent.

#### Protein Digestion and TMT Labeling

Protein digestion was performed according to the FASP procedure described by Wisniewski, Zougman et al. (Wiśniewski et al., 2009). According to the manufacturer’s instructions, the resulting peptide mixture was labeled using the 16-plex TMT reagent (Applied Biosystems, MA, United States).

#### Peptide Fractionation With Strong Cation Exchange (SCX) Chromatography

TMT labeled peptides were fractionated by SCX chromatography using the AKTA Purifier system (GE Healthcare, MA, United States). The dried peptide mixture was reconstituted and acidified with 2 ml buffer A (10 mM KH_2_PO_4_ in 25% of ACN, pH 2.7) and loaded onto a polysulfoethyl 4.6 × 100 mm column (5 µm, 200 Å, PolyLC Inc., MD, United States). The peptides were eluted at a flow rate of 1 ml/min with a gradient of 0–10% buffer B (500 mM KCl, 10 mM KH_2_PO_4_ in 25% of ACN, pH 2.7) for 2 min, 10–20% buffer B for 25 min, 20–45% buffer B for 5 min, and 50–100% buffer B for 5 min. The elution was monitored by absorbance at 214 nm. Eluting fractions were collected every 1 min. The collected fractions (about 30 fractions) were finally combined into 10 pools and desalted on C18 Cartridges (Empore™ SPE Cartridges C18, bed I. D. 7 mm, volume 3 ml, Sigma, United States). Each fraction was concentrated by vacuum centrifugation and reconstituted in 40 µl of 0.1% (v/v) trifluoroacetic acid. All samples were stored at −80°C until LC-MS/MS analysis.

#### LC-MS/MS Analysis

Experiments were performed on a Q Exactive HF-X orbitrap mass spectrometer (Thermo Fisher Scientific, MA, United States) coupled with Easy nLC (Proxeon Biosystems, Thermo Fisher Scientific, MA, United States). 5 μl of each fraction was injected for nano-LC-MS/MS analysis. The peptide mixture (2 μg) was loaded onto a C18 reversed-phase column packed in-house with RP-C18 5 μm resin in buffer A (0.1% formic acid) and separated with a linear gradient of buffer B (80% acetonitrile and 0.1% formic acid) at a flow rate of 250 nl/min over 90 min. MS data were then acquired using a data-dependent top 20 method, dynamically choosing the most abundant precursor ions from the survey scan (300–1,800 m/z) for HCD fragmentation. Determination of the target value was based on predictive Automatic Gain Control (pAGC). Dynamic exclusion duration was 60s. The scans were obtained at the resolution of 70,000 at m/z 200, the value of resolution for high energy collisional dissociation spectra was set at 60,000 at m/z 200 (TMT 16 plex), and the width of isolation was 2 m/z. The normalized collision energy was 30 eV, and the AGC Target was 200,000. The instrument was run with peptide recognition mode enabled.

#### Proteomics Data Analysis

Mascot 2.2 and Proteome Discoverer 1.4 were used to analyze the LC-MS/MS raw data for library identification and quantitative analysis. The false discovery rate (FDR) was set as ≤0.01. Proteome Discoverer determined quantitative analysis of the ionic peak strength values of peptide segment. Differentially expressed proteins were identified through ratio-fold change (>1.2 or <0.83) as well as *p* value (<0.05) calculated with a t-test. The differentially expressed proteins were performed for future bioinformatics analysis.

#### Bioinformatics Analysis

After obtaining the differentially expressed protein, the Gene Ontology (GO) program Blast2GO (https://www. blast2go. com) was used to annotate DEPs (differential expression proteins) to create histograms of GO annotation, including cell component, biological process (BP) and molecular function (MF). The number of differential proteins included in each GO entry was counted, and the significance of differential protein enrichment in each GO entry was calculated using the hypergeometric distribution test. For pathway analysis, the differential proteins were mapped to the terms in the KEGG (Kyoto Encyclopedia of Genes and Genomes) database by using the KAAS program (http://www. genome. jp/kaas-bin/kaas_main). Protein-protein interaction networks were analyzed using the publicly available program STRING (http: //string-db. org), and the minimum required interaction score was set at 0.400. STRING is a database of known and predicted protein-protein interactions. The interactions include direct (physical) and indirect (functional) associations, and they are derived from four sources: the genomic context, high-throughput experiments, co-expression, and previous knowledge.

### Network Pharmacology for Predicting the Biological Contributions of Compounds Detected in SYT’s Fingerprint

Targets of compounds detected in SYT’s chemical fingerprint were collected from HERB Database (http://herb.ac.cn/). Targets for T2D were obtained from the GeneCards (https://www.genecards.org/). The OmicShare tool (https://www.omicshare.com/) was used to identify the common targets of the above compounds and T2D.

### Statistical Analysis

All experimental data were expressed as mean ± standard error of the mean (SEM) and analyzed using SPSS 25.0. Differences among all groups were determined using one-way analysis of variance with Dunnett’s C test. *p* < 0.05 was considered statistically significant.

## Results

### UPLC Fingerprint of SYT

**FIGURE 1:**
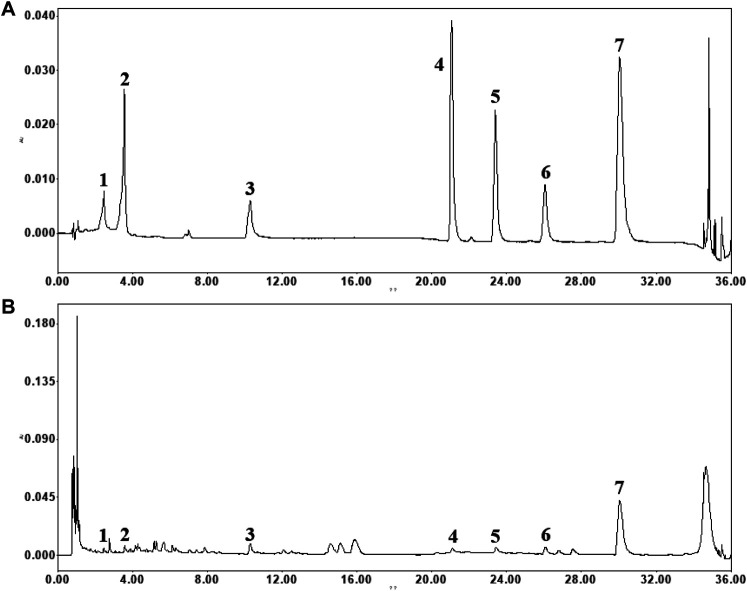
The typical chromatograms of standard compounds **(A)** and sample **(B)**. (1) danshensu, (2) 5-caffeoylquinic acid, (3) paeoniflorin, (4) astragalin, (5) rosmarinic acid, (6) lithospermic acid and (7) salvianolic acid B.

The contents of the seven well-known compounds were analyzed using the UPLC-PDA method. Representative chromatograms of the mixed seven standards and extract of formula are shown in Figure 1. The contents of the investigated analytes were as follows: 8.28 ± 0.60 mg/g for danshensu, 6.45 ± 0.06 mg/g for 5-caffeoylquinic acid, 18.15 ± 0.08 mg/g for paeoniflorin, 4.59 ± 0.04 mg/g for astragalin, 7.13 ± 0.24 mg/g for rosmarinic acid, 18.03 ± 0.43 mg/g for lithospermic acid, and 217.41 ± 3.69 mg/g for salvianolic acid B in formula (*n* = 3).

### SYT Reduces Body Weight and Viscera Weight in HFD-Induced Obese Mice

**FIGURE 2:**
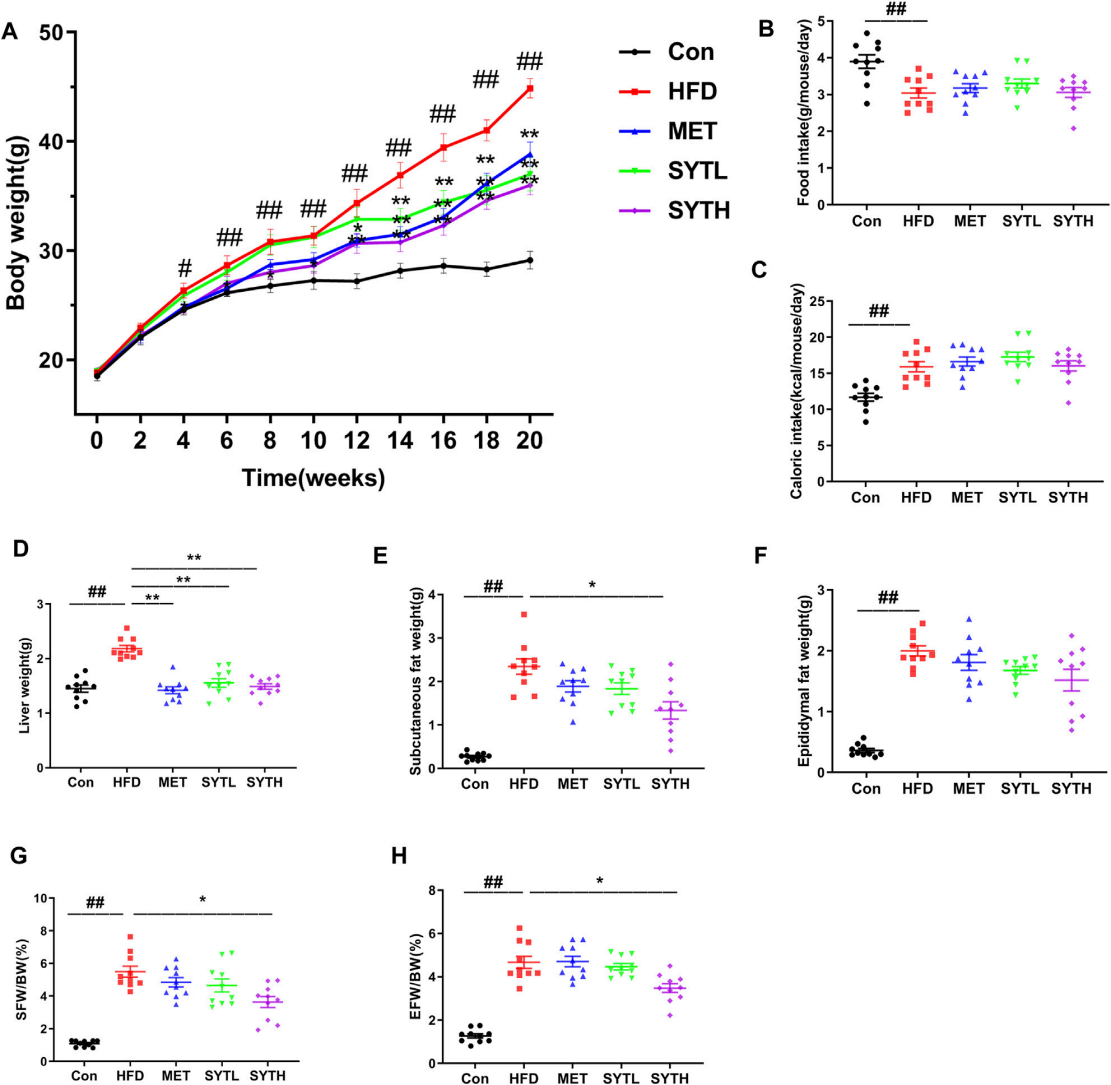
Effects of SYT on body and viscera weight, food and caloric intake in HFD mice. The bodyweight of mice was recorded regularly every week **(A)**. After treatment with SYT for 20 weeks, average daily food intake **(B)**, liver weight **(D)**, subcutaneous fat weight **(E)**, and epididymal fat weight **(F)** were measured. Average daily caloric intake **(C)**, subcutaneous fat weight/body weight **(G)**, and epididymal fat weight/body weight **(H)** were calculated. ^#^ *p* < 0.05, ^##^ *p* < 0.01 compared with Con group; ^*^ *p* < 0.05, ^**^ *p* < 0.01 compared with HFD group.

There was no difference in body weight among all groups at baseline. From 4 to 20 w, HFD significantly enhanced body weight compared to chow-fed mice (*p* < 0.05, *p* < 0.01, Figure 2A). At 20 w, HFD mice weighed 54.1% more than chow-fed mice (*p* < 0.01), although the weight was markedly alleviated by SYT and MET treatment (*p* < 0.01). Average daily food and caloric intake in chow-fed mice were 3.89 ± 0.18 g and 11.69 ± 0.54 kcal, respectively, while those in HFD mice were 3.03 ± 0.13 g and 15.93 ± 0.71 kcal (Figures 2B,C). Though these intakes noticeably changed after HFD feeding (*p* < 0.01), there was no change by SYT and MET treatment as compared to HFD mice. After that, we measured the effect of SYT on viscera weight. Weights of the liver, subcutaneous fat, and epididymal fat were significantly elevated in HFD mice, which were attenuated by MET and SYT treatment (Figures 2D–H).

### SYT Restores Glucose Tolerance and Ameliorates IR in HFD-Induced Obese Mice

**FIGURE 3:**
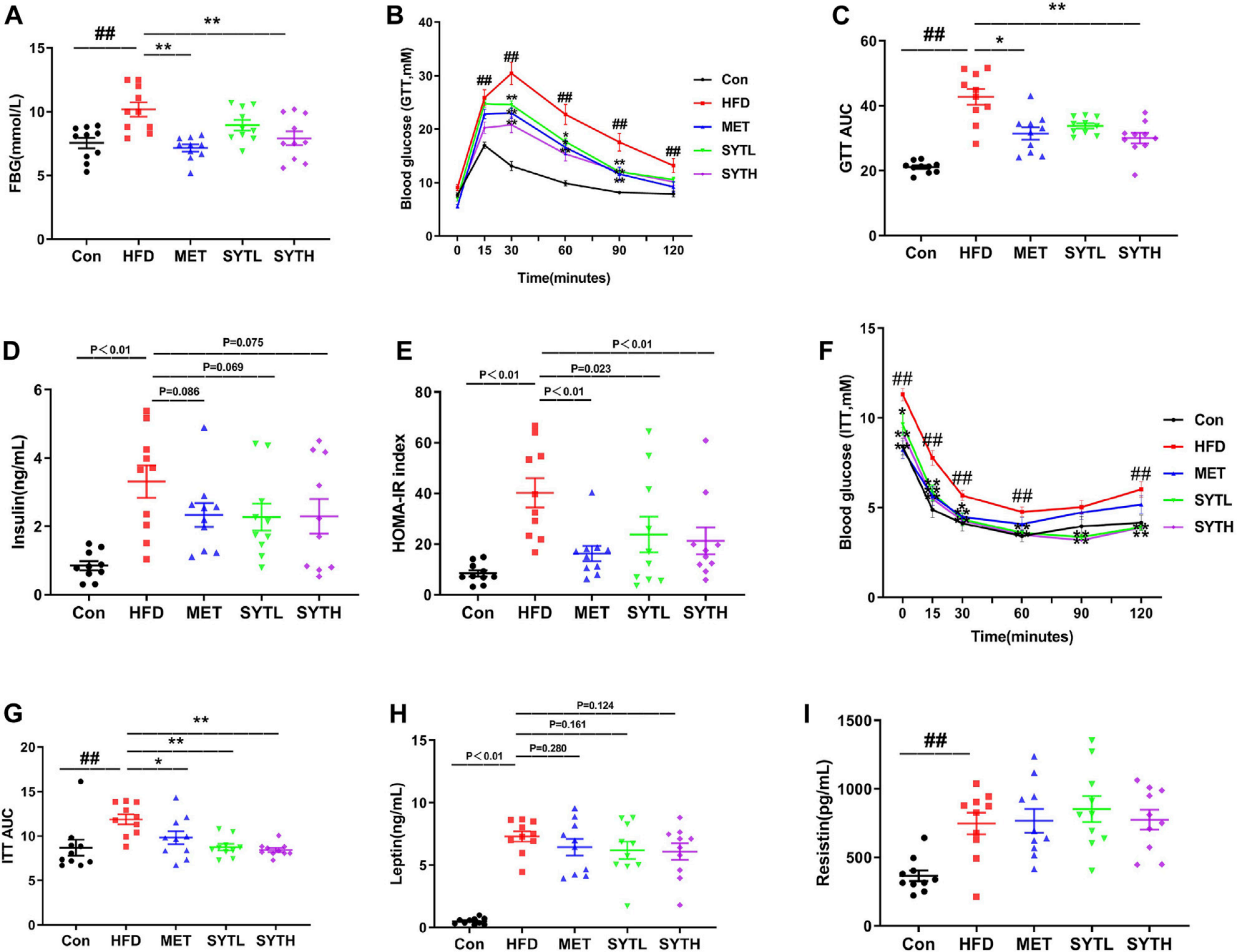
SYT restores glucose tolerance and ameliorates IR in HFD mice. After treatment with SYT for 16 weeks, fasting blood glucose **(A)** was measured. At 20 w, GTT **(B)** was measured, and AUC of GTT was represented by dot plots **(C)**. Serum insulin **(D)** and HOMA-IR index **(E)** were measured. ITT **(F)** was measured, and AUC of ITT was represented by dot plots **(F)**. Serum leptin **(H)** and serum resistin **(I)** were determined. ^##^ *p* < 0.01 compared with Con group; ^*^ *p* < 0.05, ^**^ *p* < 0.01 compared with the HFD group.

At 16 w, the fasting blood glucose (FBG) level of HFD mice was significantly increased, which was suppressed by SYT in a dose-dependent manner (Figure 3A). Notably, high dose of SYT and MET reversed the increased FBG without a difference. Then we further tested the effect of SYT on glucose tolerance by GTT at the final week. As depicted in Figures 3B,C, the blood glucose level in HFD mice was rapidly increased up to 30 min after glucose administration, whereas that level in SYT and MET treated mice exhibited a moderate increase. AUC was much lower in high-dose SYT, and MET treated mice than HFD mice (*p* < 0.05, *p* < 0.01).

IR is a key factor in the pathogenesis of metabolic syndrome; we then investigated the effect of SYT on IR by measuring fasting insulin, HOMA-IR, and ITT. As depicted in Figures 3D,E, the fasting insulin level in serum and HOMA-IR index in HFD mice was significantly higher than those in chow-fed mice (*p* < 0.01). However, increased HOMA-IR index was reversed by SYT and MET treatment (*p* < 0.05, *p* < 0.01). Consistent with the results obtained by GTT, there were moderately increased curves in ITT and smaller AUC indexes when HFD mice were treated with SYT and MET (*p* < 0.05, *p* < 0.01, Figures 3F,G). Furthermore, HFD mice treated with SYT and MET displayed minor falls in leptin compared to untreated HFD mice (Figure 3H). However, no significant difference in resistin levels was found between HFD and SYT (Figure 3I). In general, the above results indicated that SYT restored glucose tolerance and enhanced systemic insulin sensitivity.

### SYT Alleviates Lipid Accumulation in Liver and Adipose Tissue and Reduces Circulatory Inflammatory Cytokines in HFD-Induced Obese Mice

**FIGURE 4:**
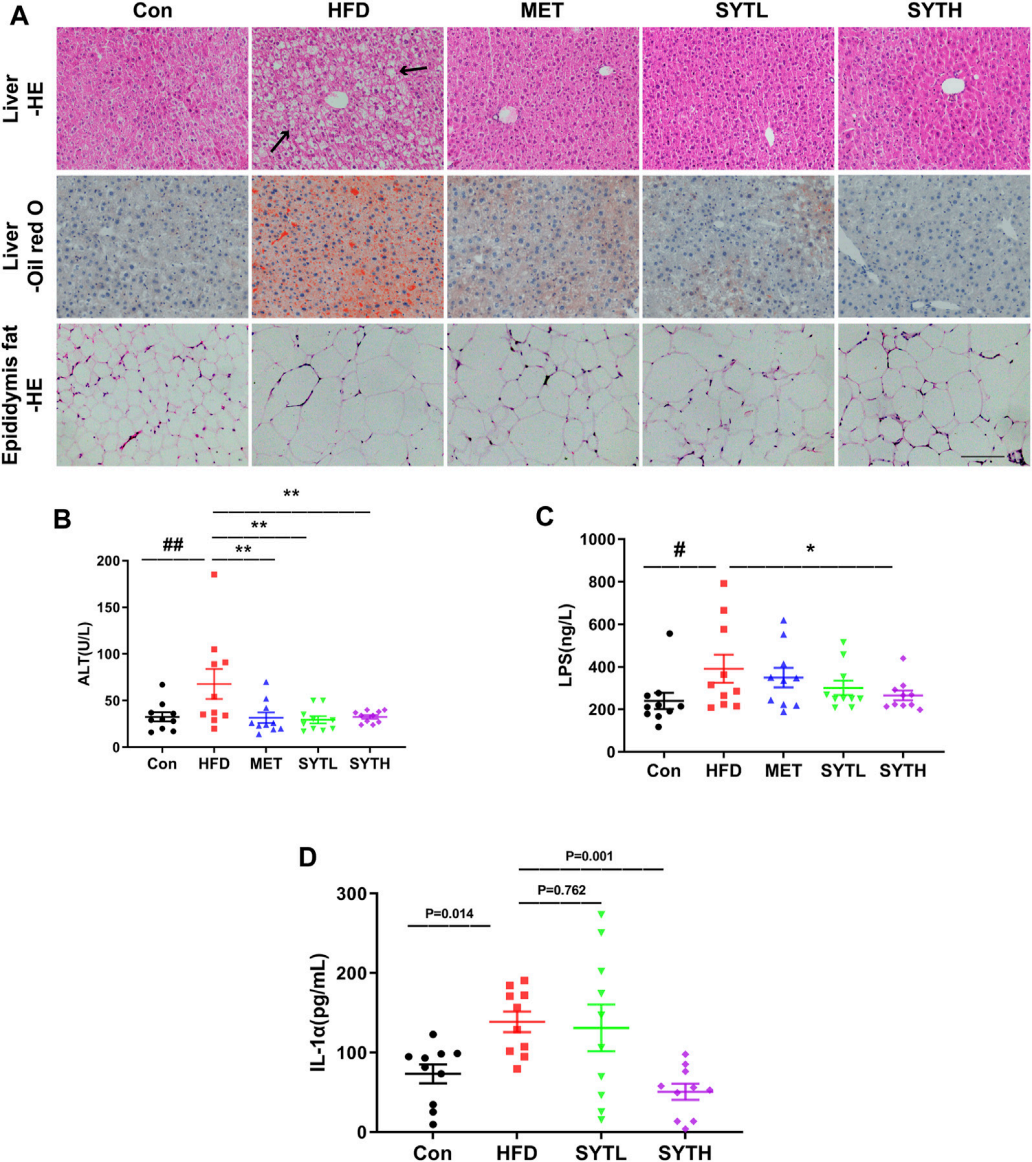
SYT alleviates hepatic steatosis and reduces circulatory inflammatory cytokines in HFD mice. After treatment with SYT for 20 weeks, serum samples, liver tissues, and epididymal fat were obtained from all the mice. Liver and epididymal fat tissues were stained with HE. Liver tissue was stained with oil red O. Scale bar = 200 μm **(A)**. The levels of serum ALT **(B)**, LPS **(C)**, and IL-1α **(D)** were measured. ^#^ *p* < 0.05, ^##^ *p* < 0.01 compared with Con group; ^*^ *p* < 0.05, ^**^ *p* < 0.01 compared with the HFD group.

To investigate the effect of SYT on hepatic steatosis, HE and oil red O staining were performed. In chow-fed mice, hepatic cords were radially arranged around a central vein with a few lipid droplets. However, significant histological abnormalities, including fat deposition and inflammatory cell infiltration, could be observed in the liver tissue of HFD mice, which were reversed by SYT treatment (Figure 4A). Furthermore, SYT treatment reversed the enhancement of adipocyte volume in the epididymis induced by HFD (Figure 4A). Consistently, the significantly increased ALT level by HFD was dramatically attenuated by SYT and MET treatment (*p* < 0.01, Figure 4B), which indicated that SYT could reduce liver injury induced by HFD. We then examined circulatory inflammatory cytokines. As shown in Figures 4C,D, HFD led to increased serum levels of LPS and IL-1α (*p* < 0.05), which were attenuated by SYT treatment in a dose-dependent manner. Especially, high-dose of SYT significantly suppressed the increased serum levels of LPS and IL-1α (*p* < 0.05, *p* < 0.01).

### SYT Improves Serum Lipid Metabolism by Lipidomics Analysis

**FIGURE 5:**
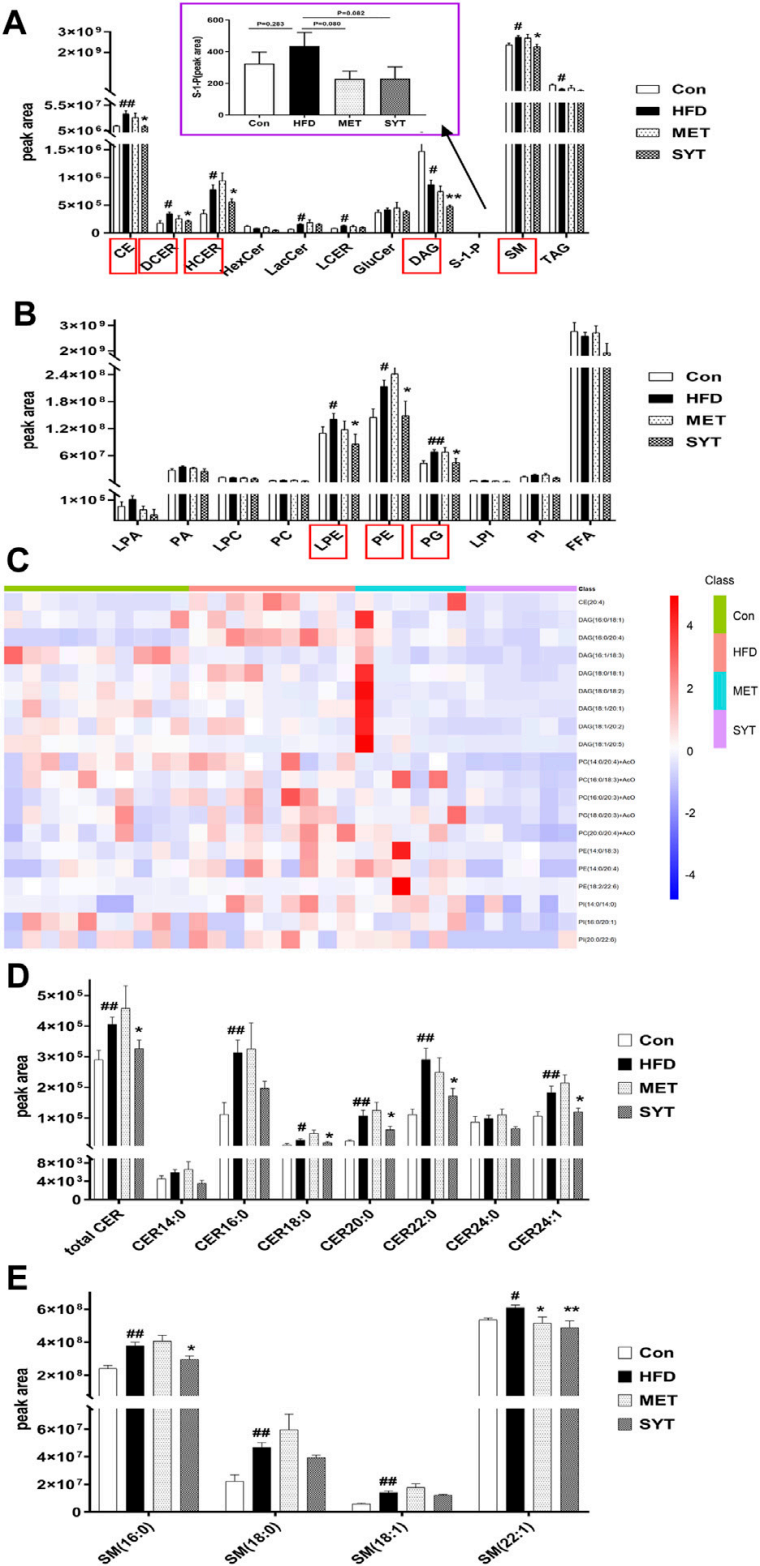
SYT improves serum lipid metabolism in HFD mice by lipidomics analysis based on UHPLC-MS. The intensity of different serum lipid compositions in positive ion mode **(A)**. The intensity of different serum lipid compositions in negative ion mode **(B)**. Heat map of TOP 20 significantly differential lipid species between SYT group and HFD group **(C)**. Alterations in serum ceramide (Cer) compositions **(D)**. Alterations in serum sphingomyelin (SM) compositions **(E)**. ^#^ *p* < 0.05, ^##^ *p* < 0.01 compared with Con group; ^*^ *p* < 0.05, ^**^ *p* < 0.01 compared with HFD group.

To study the effect of SYT on lipid metabolism in HFD mice, we analyzed the differences in lipid composition based on the lipidomics approach. Since most findings in SYTH group were significant, we focus on the effects of high dose SYT in subsequent studies. Herein, we identified and quantified serum lipids by UHPLC-MS techniques. To obtain serum lipid distribution among all groups, principal components analysis (PCA) was performed and shown in Supplementary Figure S1A. According to the PCA score plot, the lipid metabolic patterns of mice behaved differently among the four groups. Then we applied supervised PLS-DA analyses to confirm the differences among all the groups (Supplementary Figure S1B). It was observed that HFD mice clearly distinguished from chow-fed mice, while SYT (0.4 g/kg)-treated mice separated away from HFD mice and aggregated close to chow-fed mice, suggesting that SYT treatment improved the disordered metabolisms toward normalcy. In the OPLS-DA plot, the variation of metabolites could be found through observing the phase (positive or negative) between different groups (Supplementary Figure S1C–E). Moreover, a total of 21 lipid species were identified (Figures 5A,B). Compared with those in the Con group, increased cholesteryl ester (CE), dihydroceramide (DCER), hydroceramide (HCER), lactosylceramide (LacCer), lactosylceramides (LCER), sphingomyelin (SM), Lyso-PE (LPE), phosphatidylethanolamines (PE), and phosphatidylglycerols (PG) levels were detected in the HFD group (*p* < 0.05, *p* < 0.01), suggesting a marked disturbance of lipid metabolism in HFD mice. As expected, SYT caused a significant reduction in CE, DCER, HCER, diacylglycerols **(**DAG), SM, LPE, PE, and PG levels (*p* < 0.05, *p* < 0.01).

Furthermore, the heat map (Figure 5C) revealed TOP 20 significantly differential lipid species between SYT group and HFD group. As depicted, the HFD mice showed significantly higher levels of CE (20:4), DAG (16:0/18:1, 16:0/20:4, 16:1/18:3, 18:0/18:1, 18:0/18:2, 18:1/18:1, 18:1/18:2, 18:1/18:5), PC (14:0/20:4, 16:0/18:3, 16:0/20:3, 18:0/20:3, 20:0/20:4), PE (14:0/18:3, 14:0/20:4, 18:0/22:6), and phosphatidylinositols (PI) (14:0/14:0, 16:0/20:1, 20:0/22:6) compared to chow-fed mice, whereas these molecules were decreased by SYT treatment (*p* < 0.05, *p* < 0.01). In addition, MET treatment significantly increased PC (18:0/18:1) concentration 2.18 times (13.03 × 10^4^ ± 7.68 × 10^4^ vs. 5.97×10^4^ ± 3.41 × 10^4^).

Several studies have demonstrated that some species of ceramides and sphingomyelins are elevated in experimental models of HFD. Increased levels of ceramides (CER), especially CER (16:0), CER (18:0), CER (20:0), CER (22:0), CER (24:0), and CER (24:1) (Shah et al., 2008; Boini et al., 2010), as well as sphingomyelins, especially SM (16:0), SM (18:0) and SM (18:1) (Park et al., 2013), were found in plasma and organs such as liver, skeletal muscle, and heart, which contributes to insulin resistance. Therefore, in the study of lipidomics, we focused on the changes in the above lipids. As shown in Figures 5D,E, total CER, CER (18:0), CER (20:0), CER (22:0), CER (24:1), SM (16:0), and SM (22:1) in HFD mice were significantly higher than those in chow-fed mice, which were decreased by SYT administration remarkably (*p* < 0.05, *p* < 0.01).

### SYT Inhibits Key Proteins Regulating Lipogenesis in HFD-Induced Obese Mice

**FIGURE 6:**
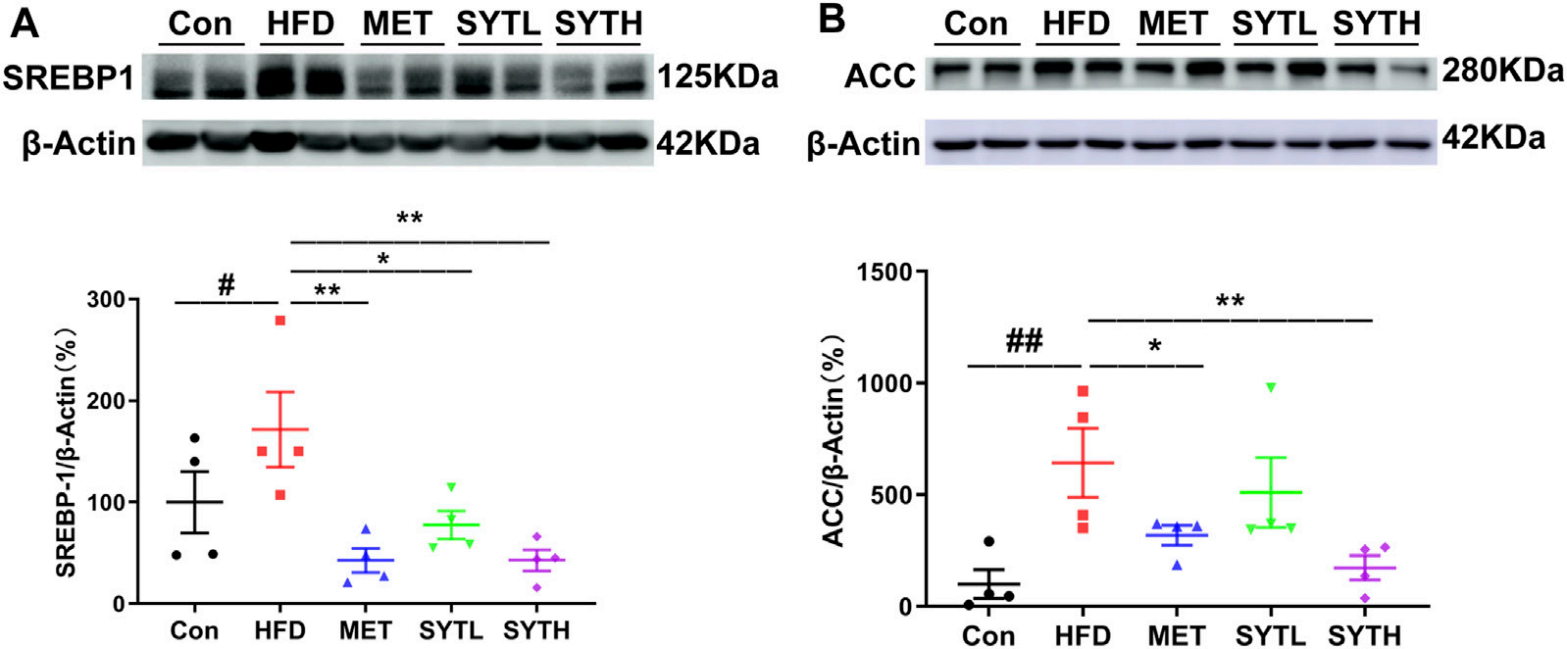
SYT downregulates protein levels of SREBP1 and ACC in HFD mice. After treatment with SYT for 20 weeks, liver tissues were obtained from all the mice. Protein levels of SREBP1 **(A)**, ACC **(B)** in the liver tissues were detected by western blot. ^#^ *p* < 0.05, ^##^ *p* < 0.01 compared with Con group; ^*^ *p* < 0.05, ^**^ *p* < 0.01 compared with HFD group.

Since SYT alleviated hepatic steatosis and improved lipid metabolism, we assayed protein levels of SREBP1 and ACC in the liver due to the core role of SREBP1 and ACC in lipogenesis regulation. As shown in Figures 6A,B, protein levels of SREBP1 and ACC were significantly higher in HFD-treated liver than in chow-fed counterparts (*p* < 0.05, *p* < 0.01). High-dose SYT caused a significant decrease in SREBP1 and ACC protein levels, which indicated that it inhibits lipogenesis by downregulating SREBP1 and ACC protein levels (*p* < 0.01).

### SYT Improves Lipid Metabolism via JAK-STAT Signaling Pathway by Proteomics Analysis

**FIGURE 7:**
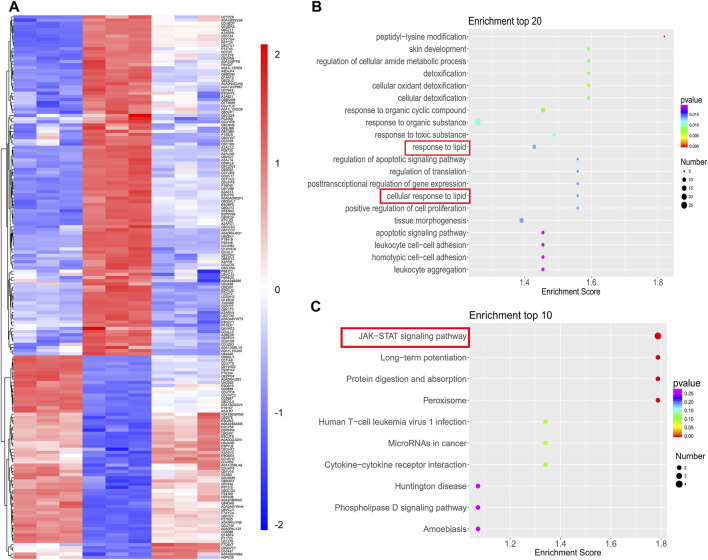
TMT-based quantification of proteomes and analysis. **(A)** Hierarchical clustering of 171 common differentially expressed proteins. **(B)** Top 20 BP enrichment terms. **(C)** Top 10 KEGG pathway terms.

To determine the exact mechanism by which SYT improved lipid metabolism abnormalities, proteomics analysis was performed. Firstly, a total of 4,824 proteins were identified based on three replicated biological analyses (Supplementary Table S1). Compared with chow-fed mice, 432 upregulated proteins and 344 downregulated proteins were identified for HFD mice, while 147 upregulated proteins and 203 downregulated proteins were identified in that of SYT treated mice compared with HFD mice (Supplementary Figure S2A). Among these comparison groups, 171 common differentially expressed proteins, including 64 upregulated proteins and 107 downregulated proteins, were identified (Supplementary Figure S2B, Figure 7A).

We further investigated these common proteins by bioinformatics analysis (Supplementary Figure S3, Figure 7B). In GO analysis, 1,343 terms in BP, 96 terms in CC, and 256 terms in MF with *p* < 0.05 were significantly enriched. Among them, response to lipid was in the top 10 of BP enrichment (Figure 7B), suggesting that SYT could modulate the biological process of lipid in HFD mice.

Next, we enriched the pathways of these differentially expressed proteins by KEGG enrichment analysis. Notably, the JAK-STAT signaling pathway was the top enriched term (Figure 7C). Additionally, in the KEGG enrichment, the pathways associated with significant differences in glucose and lipid metabolism were the JAK-STAT signaling pathway, the AGE-RAGE signaling pathway, the TNFα signaling pathway, biosynthesis of unsaturated fatty acids, fatty acid metabolism, metabolic pathways, the PPAR signaling pathway, and fatty acid degradation (Table 1). Expectedly, among them, the JAK-STAT signaling pathway contained the most differentially expressed proteins, which indicated that the regulation effect of SYT on glucose and lipid metabolism disorder might be related to the JAK-STAT signaling pathway. In the JAK-STAT signaling pathway, an increased protein level of II22ra1 was detected in HFD mice, which was significantly reversed by SYT treatment. Furthermore, we assessed the relationship among these differentially expressed proteins by STRING database. 91 nodes and 162 edges were interconnected (Supplementary Figure S4). We labeled two nodes (Il22rα1, Col3a1), consistent with GO and KEGG pathway analysis.

**TABLE 1:** The pathways associated with significant differences in glucose and lipid metabolism in the KEGG enrichment.

Pathway	Gene name
JAK-STAT signaling pathway	Il22rα1, Lif, Ep300, D630039A03Rik
AGE-RAGE signaling pathway	Col3a1
TNFα signaling pathway	Lif
biosynthesis of unsaturated fatty acids	Acaa1b
fatty acid metabolism	Acaa1b
PPAR signaling pathway	Apoa2, Acaa1b
fatty acid degradation	Acaa1b

### Biological Contributions of Compounds Detected in SYT’s Chemical Fingerprint

To further identify the bioactive compounds responsible for the observed effects of SYT, we preliminarily predicted the biological contributions of the seven well-known compounds detected in SYT’s fingerprint by network pharmacology. Firstly, targets corresponding to each compound (including danshensu, 5-caffeoylquinic acid, paeoniflorin, astragalin, rosmarinic acid, lithospermic acid, and salvianolic acid B) were collected and demonstrated in Supplementary Table S2. Then, we further screened the targets involved in the mechanism of SYT on lipid metabolism and insulin sensitivity from Supplementary Table S2. Notably, TNFα and IL-6, the targets of paeoniflorin, and IL-4, the target of rosmarinic acid, are inflammatory cytokines, which activate the JAK-STAT pathway. Moreover, PIM1, the target of astragalin, and STAT1, the target of rosmarinic acid, are components of the JAK-STAT pathway. These results suggest that paeoniflorin, rosmarinic acid, and astragalin may have regulatory roles on the JAK-STAT signaling pathway and inflammation.

Next, to further confirm the contribution of the seven compounds to the anti-T2D effects mediated by SYT, common targets shared by the compounds and T2D were identified. Expectedly, among the 52 common proteins (Supplementary Table S2), TNFα, IL-6, IL-4, and STAT1 were included. Taken together, our results demonstrated that paeoniflorin, rosmarinic acid, and astragalin might be contributing to the effect of SYT on lipid metabolism and insulin sensitivity regulation via the JAK-STAT pathway and inflammation suppression.

## Discussion

Obesity and associated T2D are growing global problems (Choi et al., 2020). Obesity elicits systemic perturbations to organismal metabolism, leading to dyslipidemia and IR (Deng et al., 2016). Clinical practice has proved that SYT effectively treats diabetes, especially for the treatment of T2D patients with hypertriglyceridemia (Liu et al., 2018). Previous preclinical studies show that SYT exhibited hypolipidemic effects (Tao et al., 2010; Nan et al., 2013). However, it is unclear on the exact mechanism by which SYT improves dyslipidemia against obesity and diabesity. Thus, this study was carried out to investigate the beneficial effects of SYT on HFD-induced obesity and diabetic mouse models, combining lipidomics and proteomics methods. Our findings show that SYT exerted its effects on reducing weight, improving IR and suppressing lipogenesis.

HFD elicits body weight gain and obesity, which is a major contributor to the onset and progression of IR. MET is an oral antihyperglycemic drug widely used to treat T2D. In our study, both SYT and MET decreased body and viscera weight. Furthermore, SYT administration dramatically improved glucose tolerance and insulin sensitivity, eventually promoting recovery from IR. Notably, SYT showed no effects on food and caloric intake, suggesting that SYT reduced body weight and improved IR by affecting the metabolic process of HFD *in vivo*.

Inflammation plays a significant role in the pathogenesis of obesity. In obese individuals, the excess lipid contents due to elevated caloric intake activate inflammatory responses systemically and locally (Boden, 2011). LPS triggers pro-inflammatory responses with increased synthesis and secretion of pro-inflammatory cytokines in blood and tissues (Sircana et al., 2018; Han et al., 2019). The IL-1 family member IL-1α is a ubiquitous and pivotal pro-inflammatory cytokine (Cavalli et al., 2021). A recent report indicates a possible role of IL-1α in promoting adiposity-induced glucose intolerance and hepatic de-novo lipogenesis (Almog et al., 2019). Here, our results demonstrate that SYT reduces circulatory inflammatory cytokines in HFD-induced obese mice. Locally, lipid accumulation and inflammatory infiltration in the liver desecrate insulin sensitivity. Feeding with HFD caused hepatocyte steatosis, demonstrating large fat vacuoles, numerous fat droplets, and infiltration of inflammatory cells. However, the administration of SYT markedly attenuated the HFD-induced hepatic steatosis. As a result, SYT lowered ALT levels in serum, indicating the protective effect on the liver.

Lipidomics is a powerful tool for the identification of possible lipid biomarkers. A growing number of studies have investigated the relationship between dysregulation in lipid metabolism and the pathogenesis of T2D (Lu et al., 2019). Our results demonstrate an increase in CE, DCER, HCER, LacCer, LCER, SM, LPE, PE, and PG and a decrease in TAG and DAG in serum of HFD mice, While the levels of CE, DCER, HCER, DAG, SM, LPE, PE, and PG were significantly downregulated by SYT treatment. Although TAG is the ubiquitous lipid, it has been reported that increased TAG levels are associated with obesity but do not appear to cause insulin resistance (Erion and Shulman, 2010; Samuel et al., 2010). However, TAG precursor DAG attracts more attention due to its role as an early predictor of IR (Taltavull et al., 2020). HFD-stimulated serum accumulation of DAG contributes to systemic IR by blocking insulin signaling pathways (Choi and Snider, 2015). We found that the administration of SYT prevented the serum accumulation of DAG, which helps to improve systemic IR and further reduce hepatic steatosis.

Phospholipids are the second largest group of lipids in the human body. The abnormal phospholipid metabolism is also associated with IR. Recently, the pivotal roles of sphingolipids in a variety of metabolic disorders have attracted growing attention. Cers are the parent structure of sphingolipids and have become the best-studied sphingolipids related to IR (Yang et al., 2018). It is well established that serum accumulation of Cers stimulated by HFD induces systemic IR by blocking insulin signaling pathways (Choi and Snider, 2015). Plasma DCER is defined as diabetes susceptibility and pre-T2D biomarker in mice and humans (Wigger et al., 2017). In addition, like Cers, the most abundant sphingolipid SM is also associated with systemic IR by impeding insulin signaling pathways (Jung et al., 2020). A large number of studies have reported that HFD accounts for an increase of many types of sphingolipids in plasma, including ceramide (C14:0, C16:0, C18:0, C20:0, C22:0, C24:0, C24:1, and total Cers) (Shah et al., 2008; Boini et al., 2010; Longato et al., 2012; Brozinick et al., 2013), SM (SM16:0, SM18:0, SM18:1, and SM22:1) (Eisinger et al., 2014), and S-1-P (Li et al., 2011; Bruce et al., 2012; Kowalski et al., 2013). Here, SYT largely prevented the serum accumulation of these sphingolipids in HFD mice. Moreover, PC and PE are the most abundant glycerophospholipids. Specific PC species with (18:0/18:1) improves glucose tolerance and insulin sensitivity in mice (Liu et al., 2013). In our study, MET increased PC (18:0/18:1) concentration 2.18 times; however, this was not affected by SYT.

SREBP1 is a transcription factor for the enzymes of *de novo* lipogenesis, including FAS and ACC. The continuous activation of SREBP1 triggers hepatic steatosis by enhancing TAG accumulation, which happens in high-calorie or high-fat diet populations. ACC accelerates lipogenesis, presenting increasing DAG and SM generation, which blocks the insulin signaling pathway. In our study, consistent with DAG and SM levels and hepatocyte steatosis, HFD induced upregulation of SREBP1 and ACC protein expressions, which could be attenuated by SYT treatment. Intriguingly, it has been reported that in the hepatic cell activated insulin signaling pathway promotes the synthesis and processing of SREBP1, leading to the maturation and nuclear translocation of SREBP1 (Zhao et al., 2020). When insulin resistance occurs, the hepatic insulin signaling pathway is inhibited, but liver lipid synthesis increases. Therefore, we hypothesized that SYT mitigated lipogenesis by modulating insulin-independent signaling pathways.

Proteomics method helps to identify biomarkers and mechanisms. Based on our results and doubts, we further detected differentially expressed proteins in the liver of HFD mice in virtue of proteomics methods. 171 common differentially expressed proteins, including 64 upregulated proteins and 107 downregulated proteins, were identified. GO enrichment analysis for these common proteins implied SYT modulated the BP response to lipid. Furthermore, KEGG pathway analysis showed that the JAK-STAT signaling pathway was the topmost and contained the most differentially expressed proteins, which indicated that the effects of SYT on HFD were related to the JAK-STAT signaling pathway. In the classical JAK-mediated pathway, when extracellular cytokines bind to its receptor, phosphorylated JAK initiates STAT recruitment and phosphorylation—activated STAT, then translocate to the nucleus to bind DNA elements and regulate transcription of associated genes (Salas et al., 2020). JAK/STAT signaling pathway plays a chief part in hepatic metabolism. Disruption of JAK/STAT signaling pathways eventually result in hepatic steatosis and insulin resistance. Reports have proved the lipid-regulating effect of STAT3. Mice lacking the STAT3 activity showed increased liver lipid accumulation and upregulated expression of SREBP1 after being fed with the HFD (Kinoshita et al., 2008; Sachithanandan et al., 2010). In our present study, as one of the cytokine receptors, interleukin-22 receptor subunit alpha-1 (Il22rα1) was increased in the HFD liver, while SYT significantly attenuated this enhancement.

We further predicted the biological contributions of the seven well-known compounds detected in SYT’s fingerprint by network pharmacology. Our study provided a hint that astragalin and rosmarinic acid might regulate the JAK-STAT pathway by targeting PIM2 and STAT1, respectively, while paeoniflorin and rosmarinic acid were likely to regulate inflammatory disorder by targeting TNFα, IL-6, and IL-4 during T2D. However, the bioactive compounds in SYT and their actions need to be explored in future pharmacological evaluation-based studies.

## Conclusion

**FIGURE 8:**
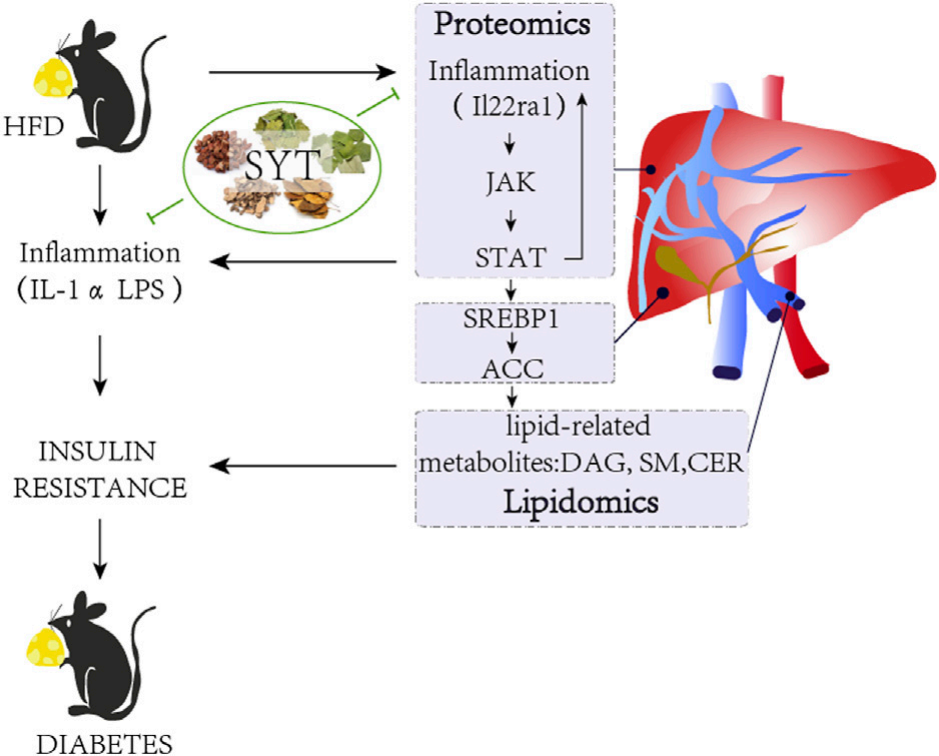
Schematic diagram describing the mechanism of SYT on HFD-induced obesity mice.

Several inhibitors of JAKs show the potential to affect pro-inflammatory cytokine-dependent pathways, and some of them are in clinical development for the treatment of sterile inflammatory diseases. Our results have indicated that SYT possesses the potential of improving obesity-associated T2D through the regulation of lipid-related metabolites via the JAK/STAT signaling pathway in the liver (Figure 8).

## Data Availability

The original contributions presented in the study are included in the article/Supplementary Material, further inquiries can be directed to the corresponding author. The data presented in the study are deposited in the ProteomeXchange Consortium repository, accession number PXD028317.

## Glossary

ACC: Acetyl-CoA Carboxylase
ALT: Alanine Transaminase
AUC: Area Under the Curve
BP: Biological Process
CC: Cellular Component
CE: Cholesteryl ester
Cer: Ceramides
Con: Basal diet group
DAG: Diacylglycerols
DCER: Dihydroceramide
EFW: Epididymal Fat Weight
FFA: Free Fatty Acids
GluCer: Glucosylceramides
GTT: Glucose Tolerance Test
HCER: Hydroceramide
HE: Hematoxylin and Eosin
HexCer: Hexosylceramide
HFD: High-Fat Diet
HOMA-IR: Homeostasis Model Assessment of Insulin Resistance
IR: Insulin Resistance
ITT: Insulin Tolerance Test
Kcal: Kilocalorie
LacCer: Lactosylceramide
LCER: Lactosylceramides
LPA: Lyso-PA
LPC: Lyso-PC
LPE: Lyso-PE
LPI: lysophosphatidylinositol
LW: Liver Weight
MET: Metformin group
MF: Molecular Function
PA: Phosphatidic acids
PC: Phosphatidylcholines
PE: Phosphatidylethanolamines
PE-O: Plasmanyl glycerophosphatidylethanolamine
PE-P: Plasmenyl glycerophosphatidylethanolamine
PG: Phosphatidylglycerols
PI: Phosphatidylinositols
SFW: Subcutaneous Fat Weight
SM: Sphingomyelin
S-1-P: sphingosine-1-phosphate
SREBP-1: Sterol Regulatory Element-binding Protein-1
SYT: Sanye Tablet
SYTL: HFD with 0.2 g/kg SYT
SYTH: HFD with 0.4 g/kg SYT
TAG: Triacylglycerols
TC: Total Cholesterol
T2D: Type 2 Diabetes
