# Supplementary figures and images for: Sanye Tablet Ameliorates Insulin Resistance and Dysregulated Lipid Metabolism in High-Fat Diet-Induced Obese Mice
Source: Front Pharmacol. 2021 Sep 29;12:713750. doi: 10.3389/fphar.2021.713750 (PMC8511530; doi:10.3389/fphar.2021.713750)

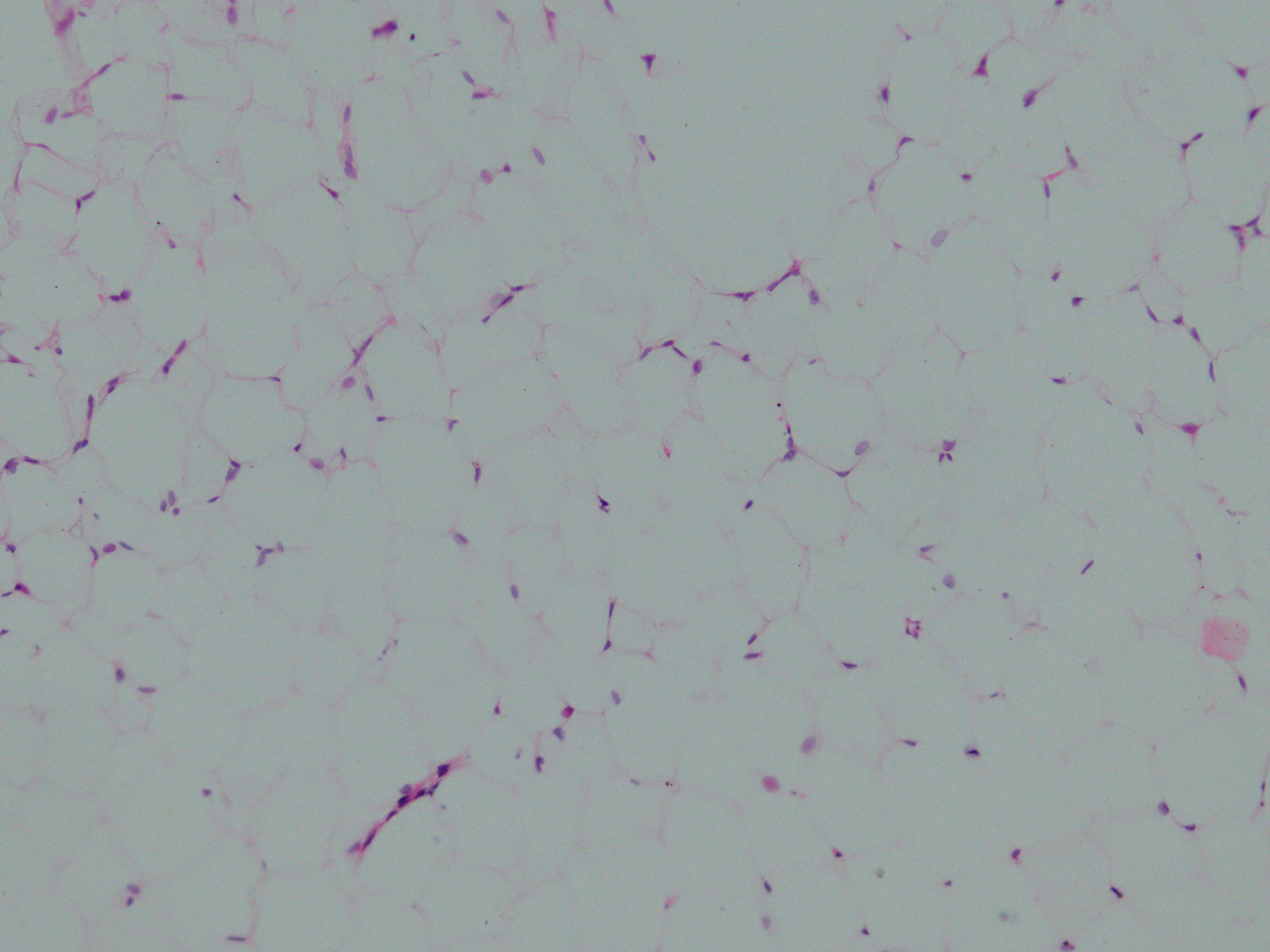

Supplement: Supplementary file 1 [file DataSheet3.ZIP › Epididymis fat HE/C.tif]

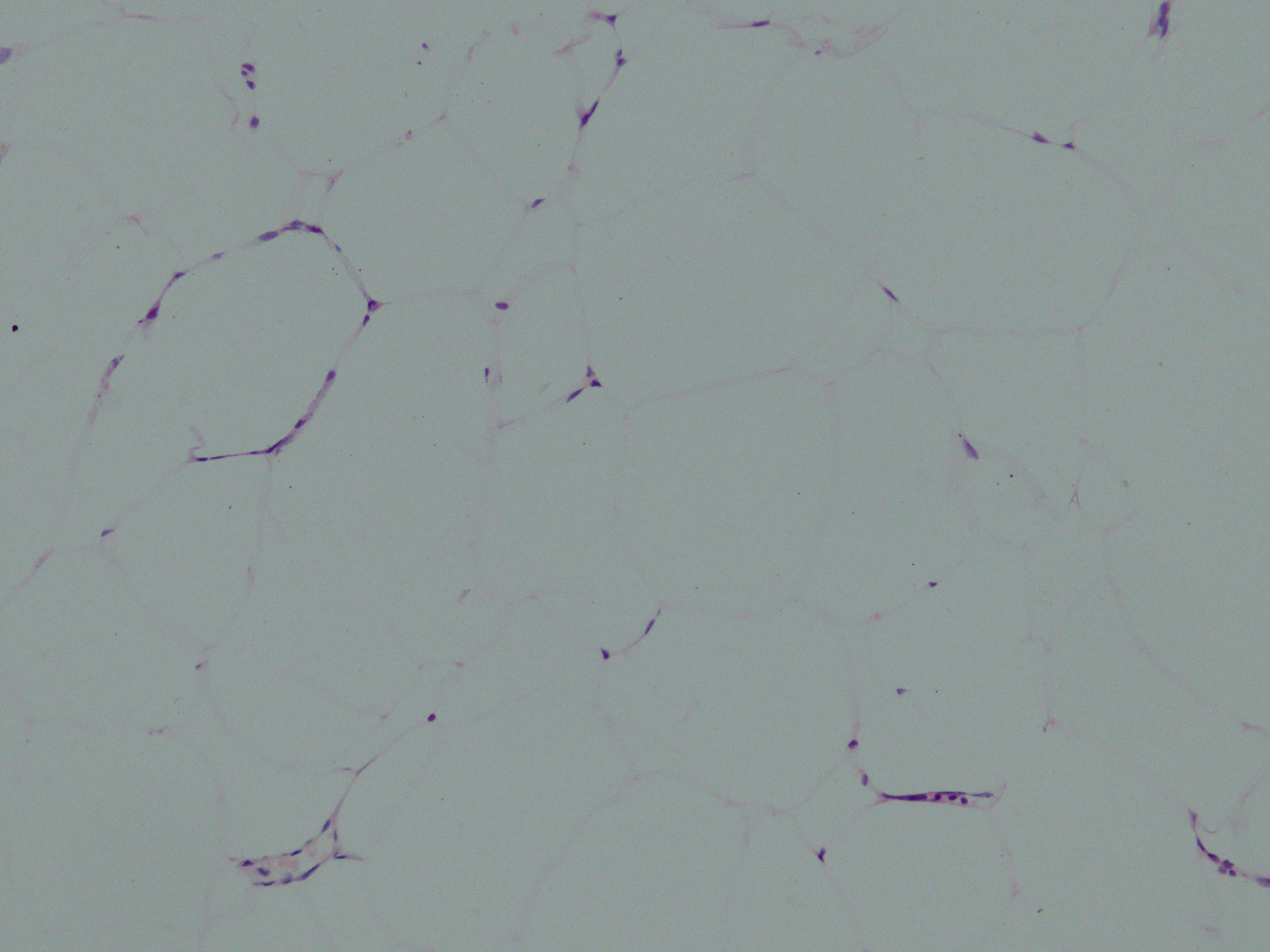

Supplement: Supplementary file 1 [file DataSheet3.ZIP › Epididymis fat HE/HFD.tif]

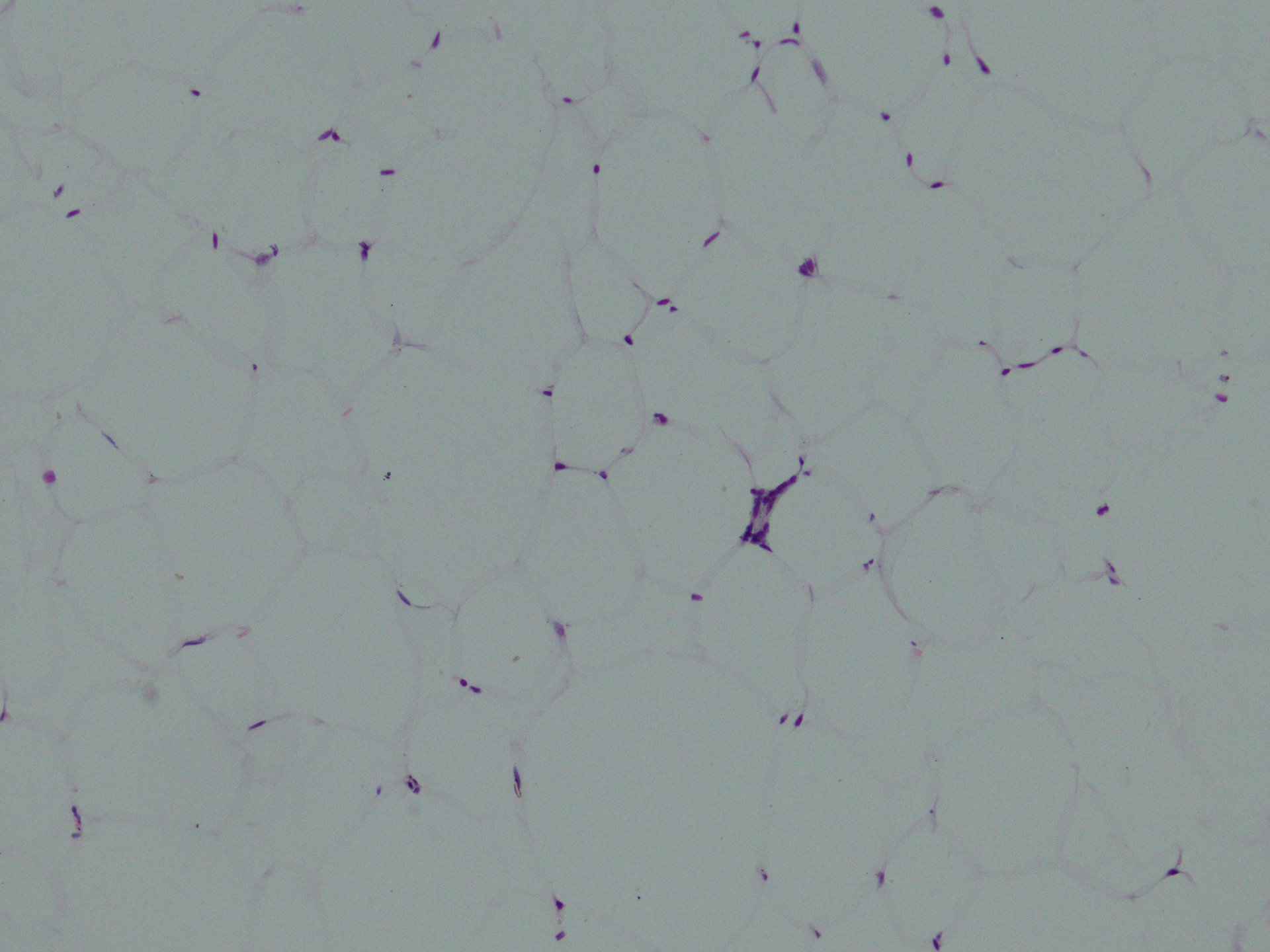

Supplement: Supplementary file 1 [file DataSheet3.ZIP › Epididymis fat HE/MET.tif]

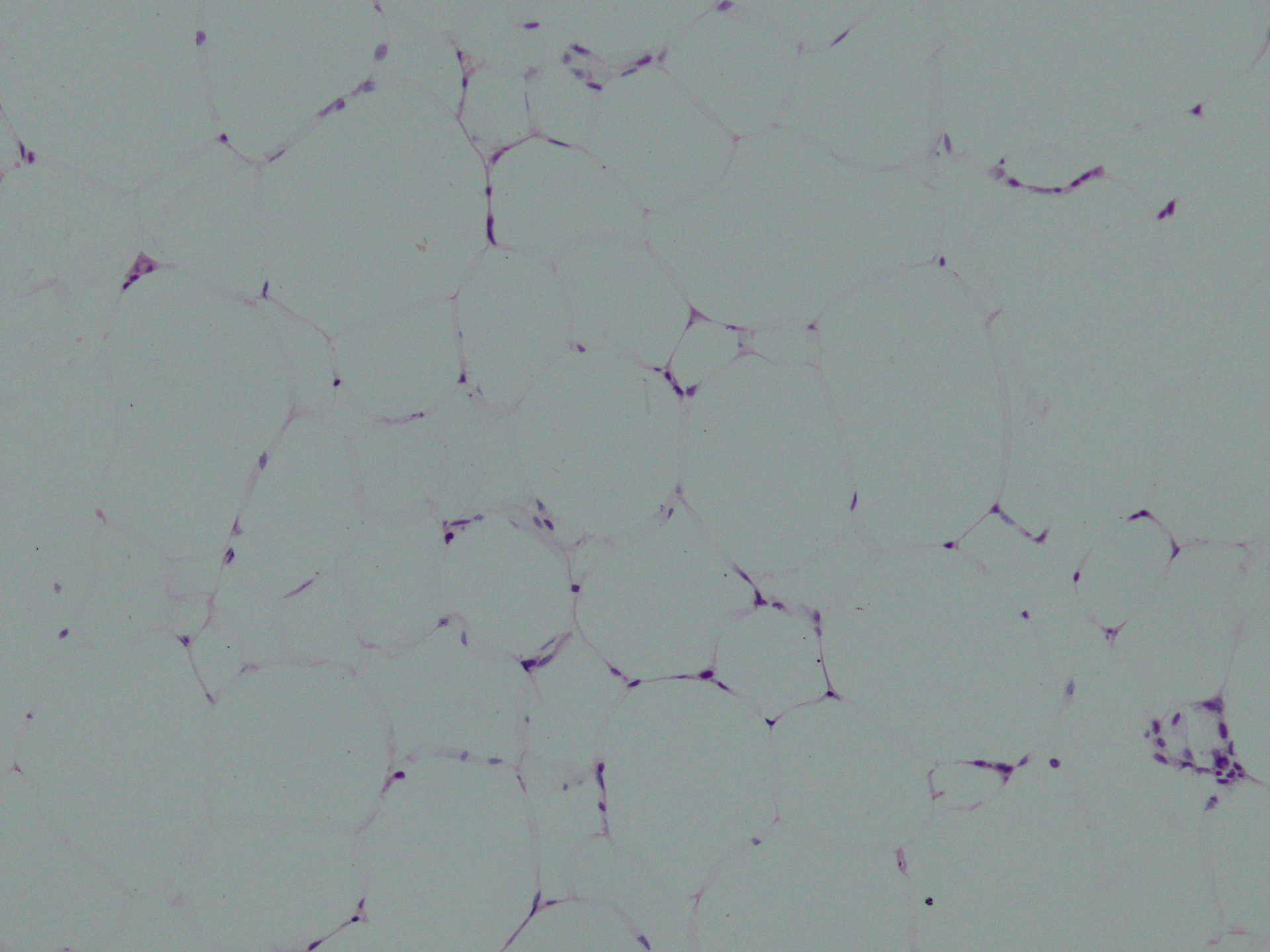

Supplement: Supplementary file 1 [file DataSheet3.ZIP › Epididymis fat HE/SYTH.tif]

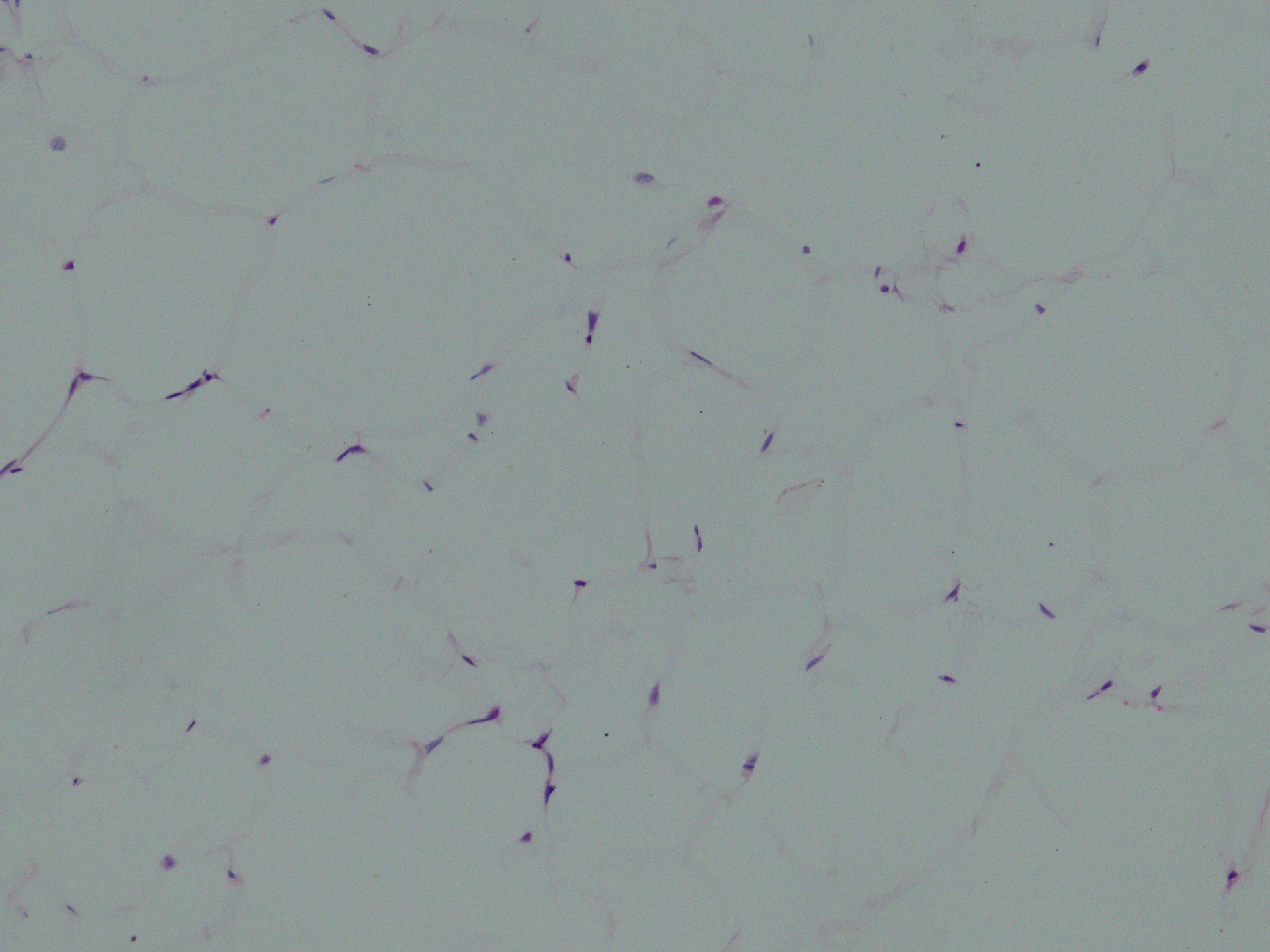

Supplement: Supplementary file 1 [file DataSheet3.ZIP › Epididymis fat HE/SYTL.tif]

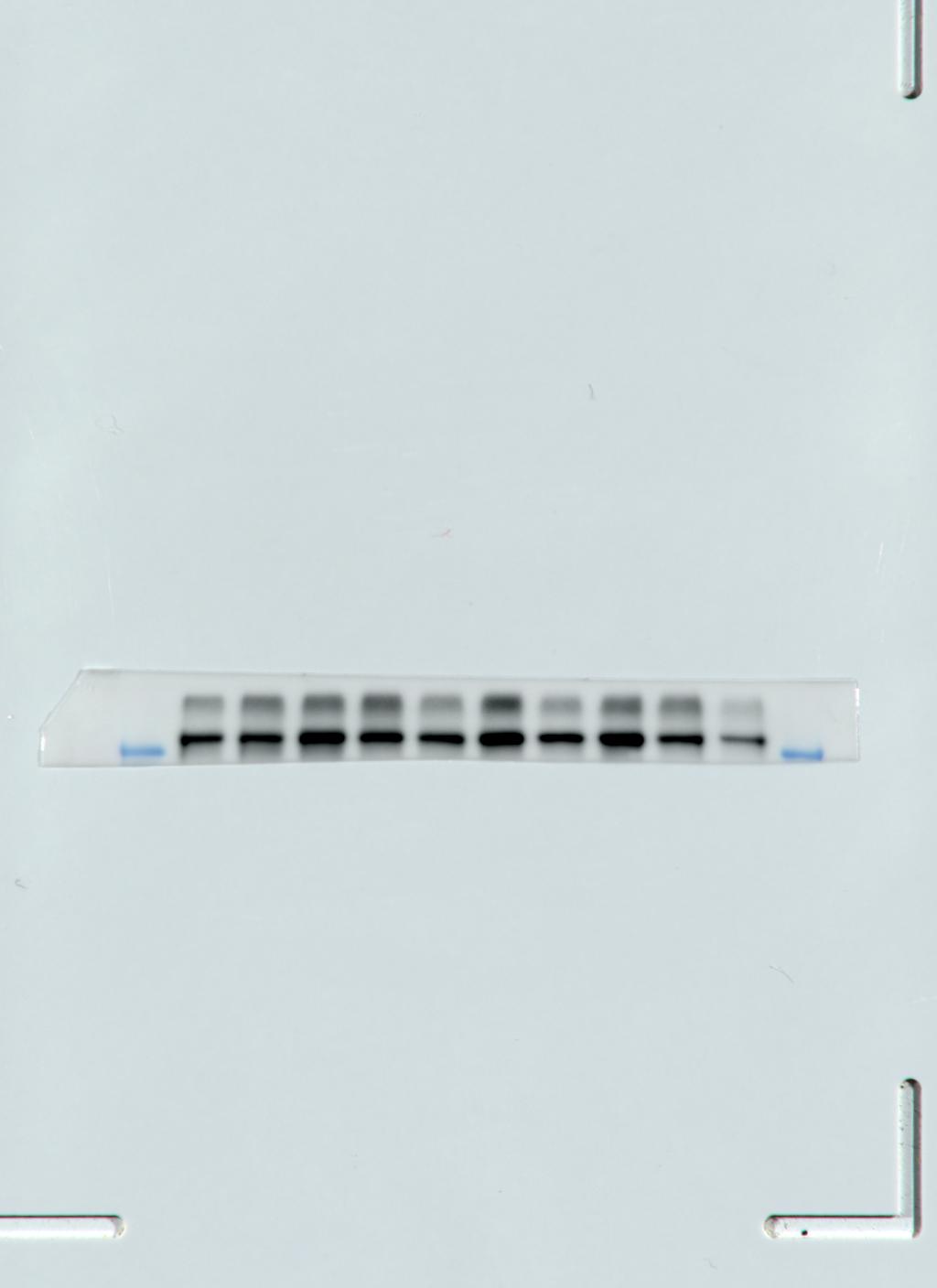

Supplement: Supplementary file 2 [file DataSheet8.ZIP › western blot/ACC/ACC.jpg]

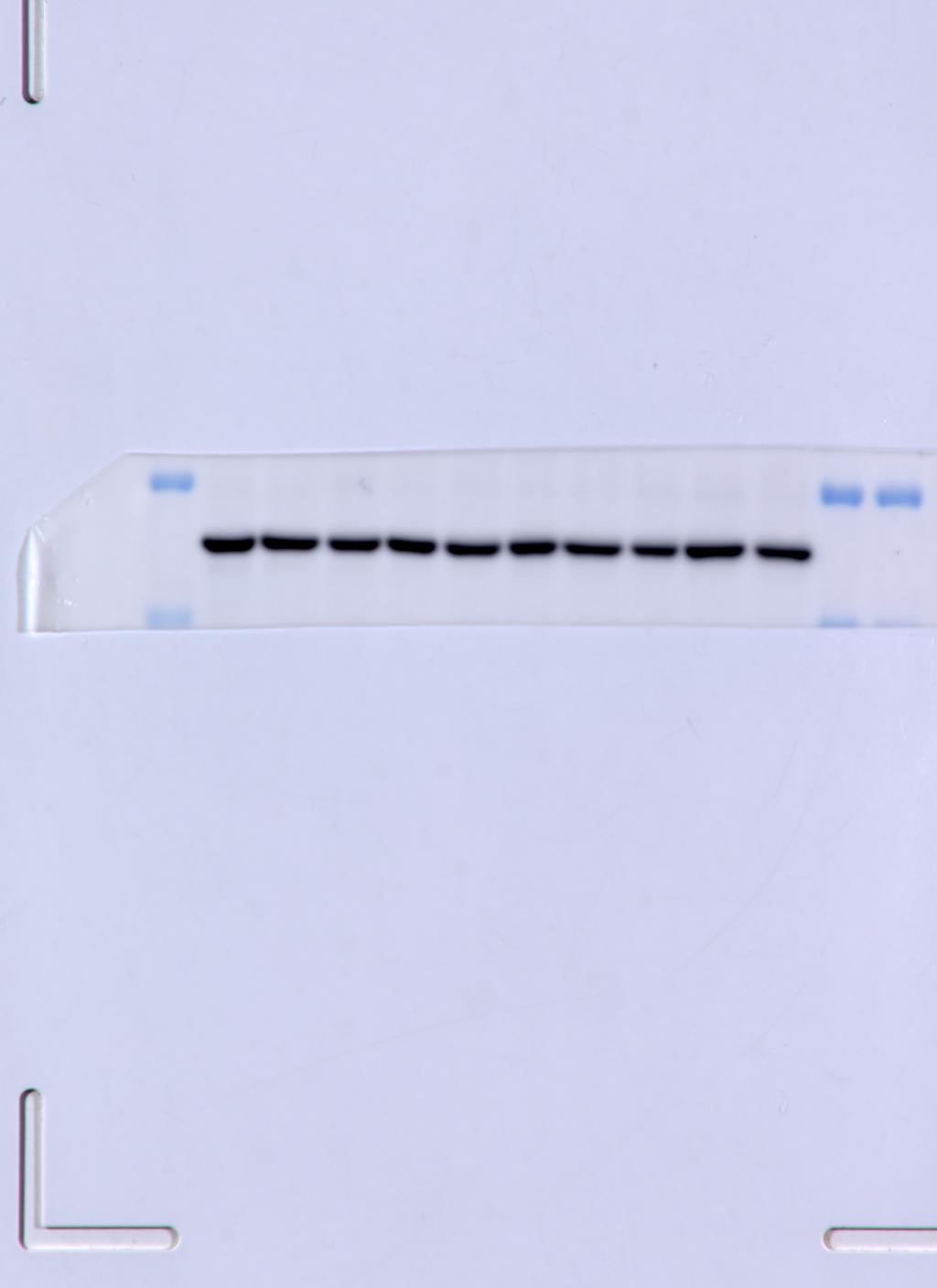

Supplement: Supplementary file 2 [file DataSheet8.ZIP › western blot/ACC/a┬-Actin.jpg]

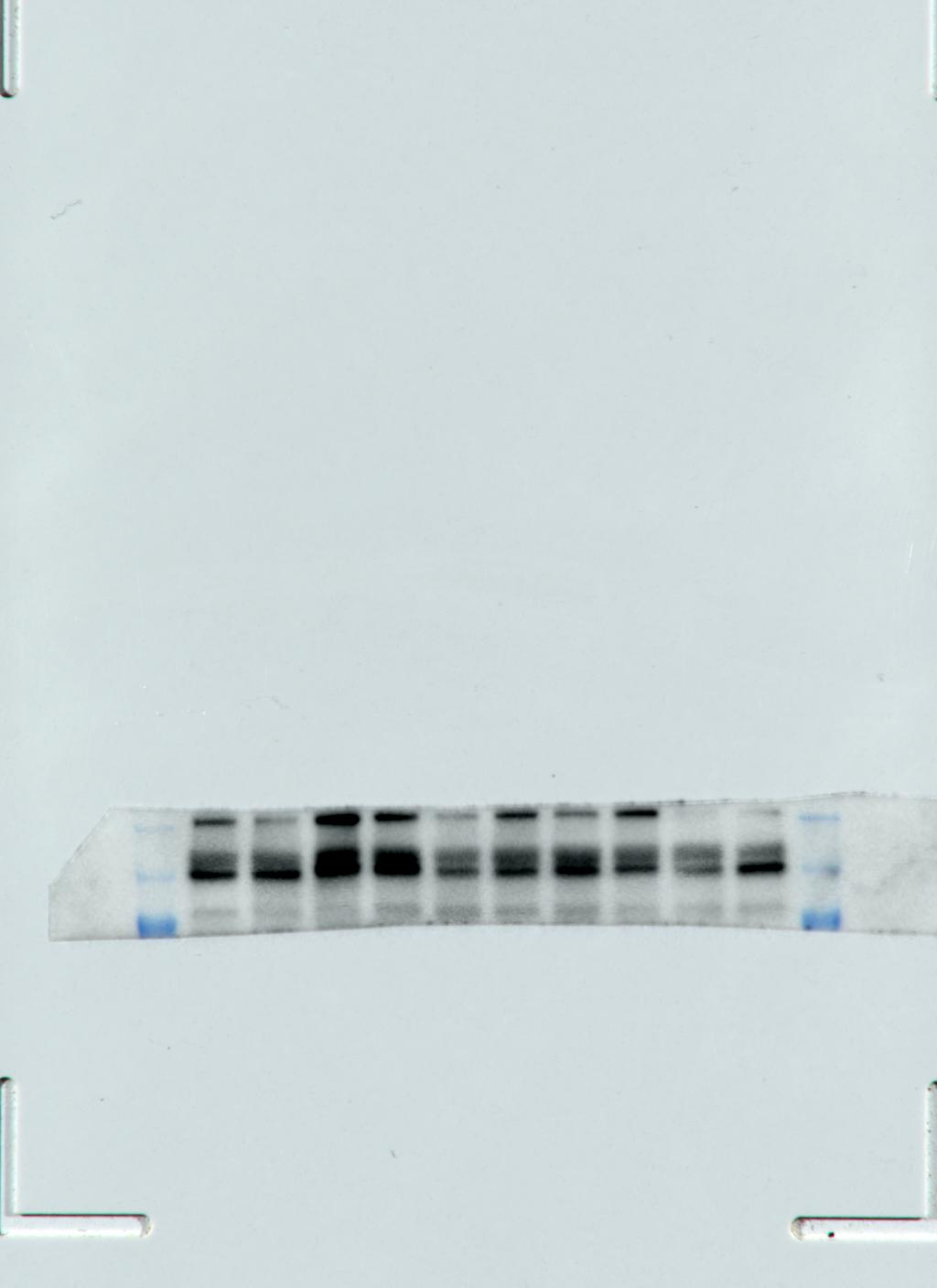

Supplement: Supplementary file 2 [file DataSheet8.ZIP › western blot/SREBP1/SREBP1.jpg]

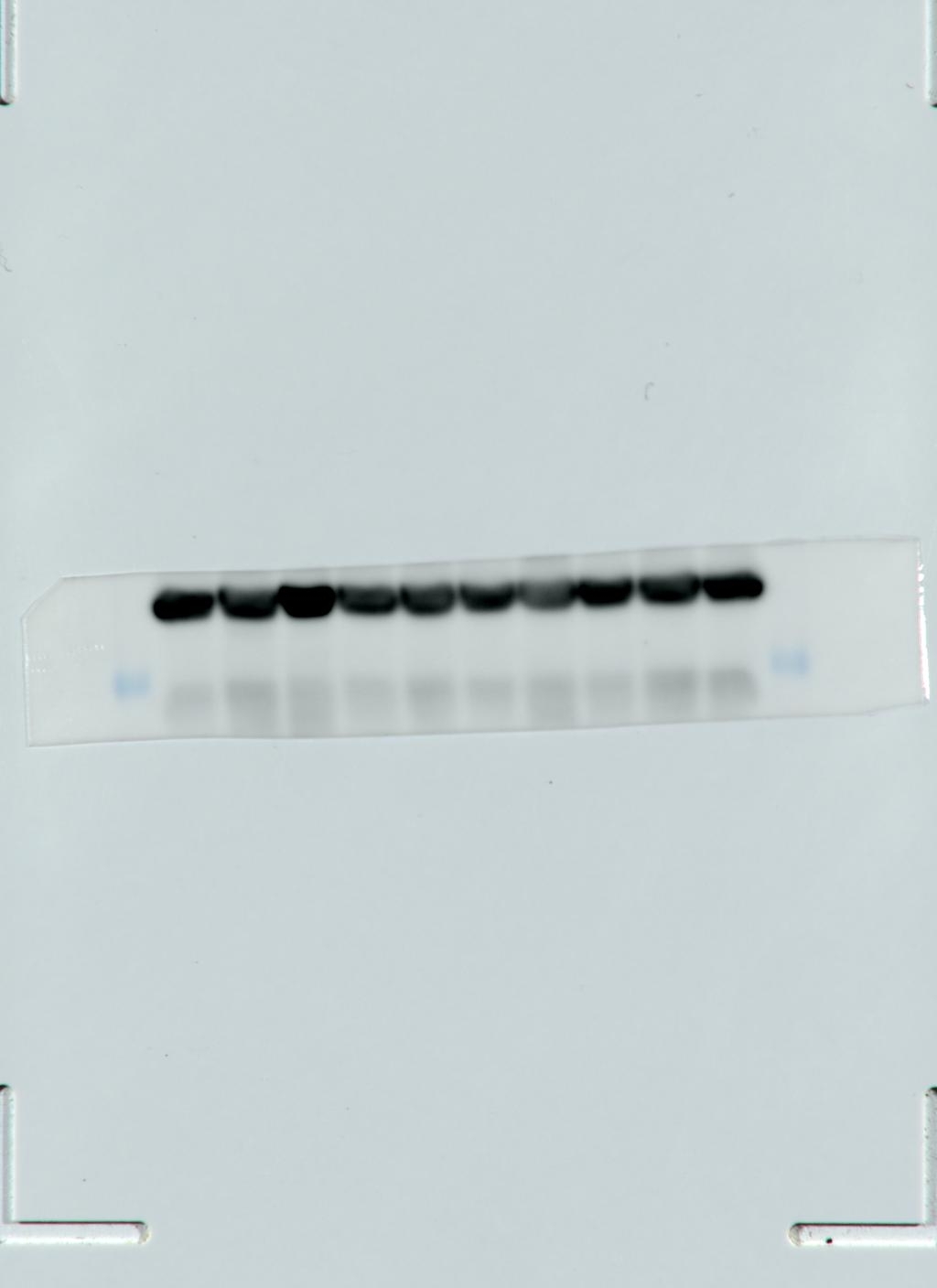

Supplement: Supplementary file 2 [file DataSheet8.ZIP › western blot/SREBP1/a┬-Actin.jpg]

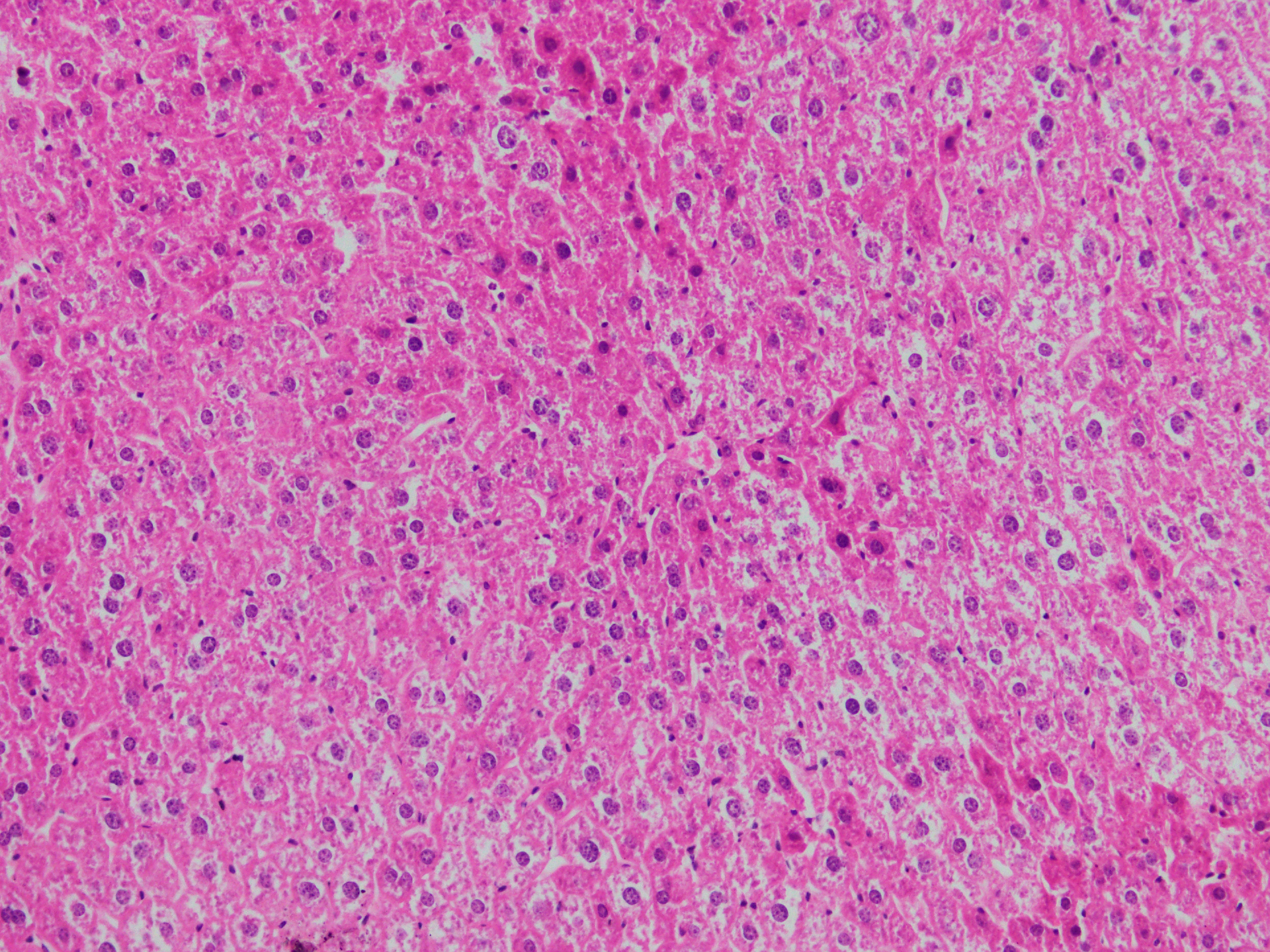

Supplement: Supplementary file 4 [file DataSheet4.ZIP › Liver HE 1/C.tif]

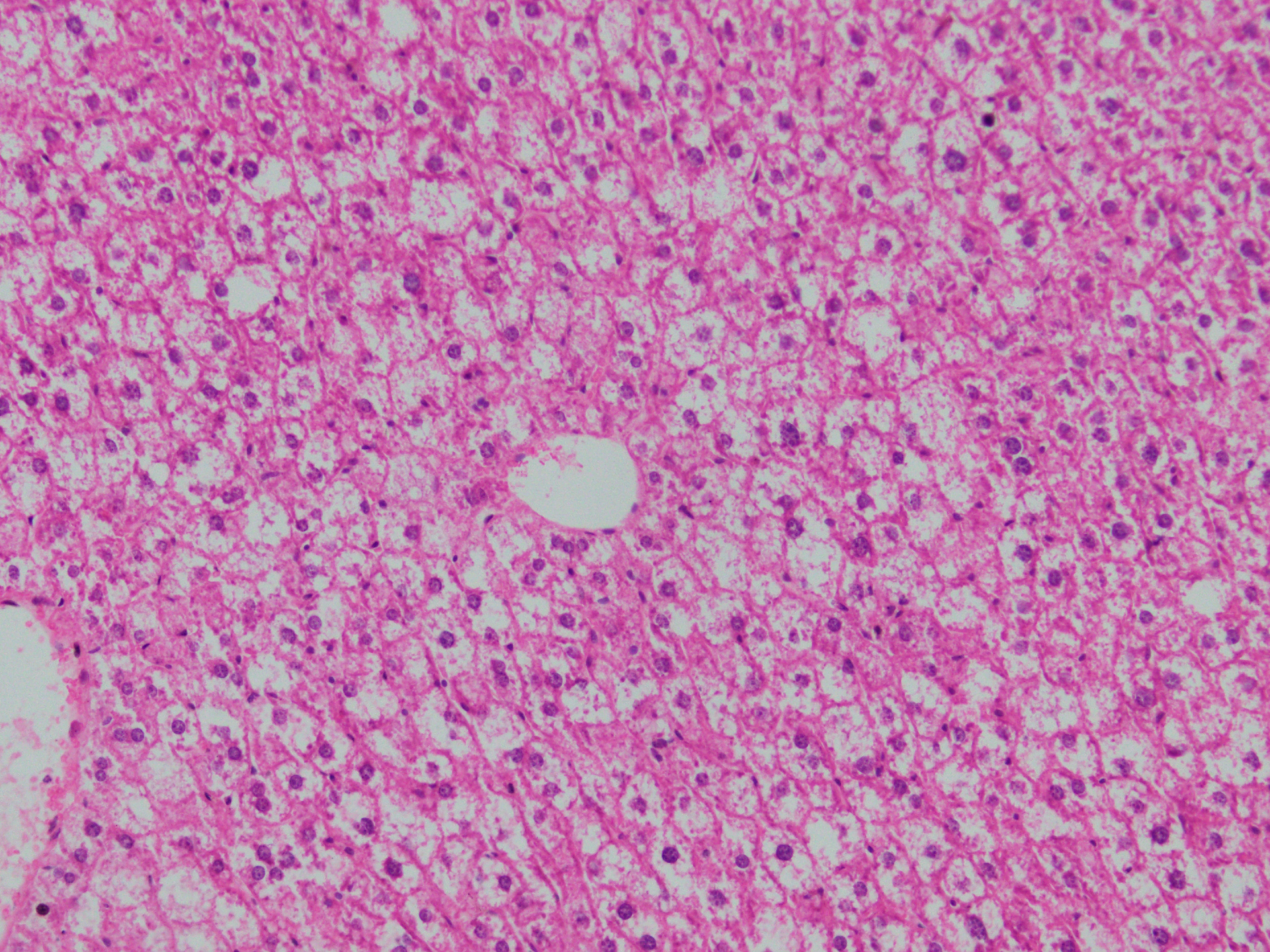

Supplement: Supplementary file 4 [file DataSheet4.ZIP › Liver HE 1/HFD.tif]

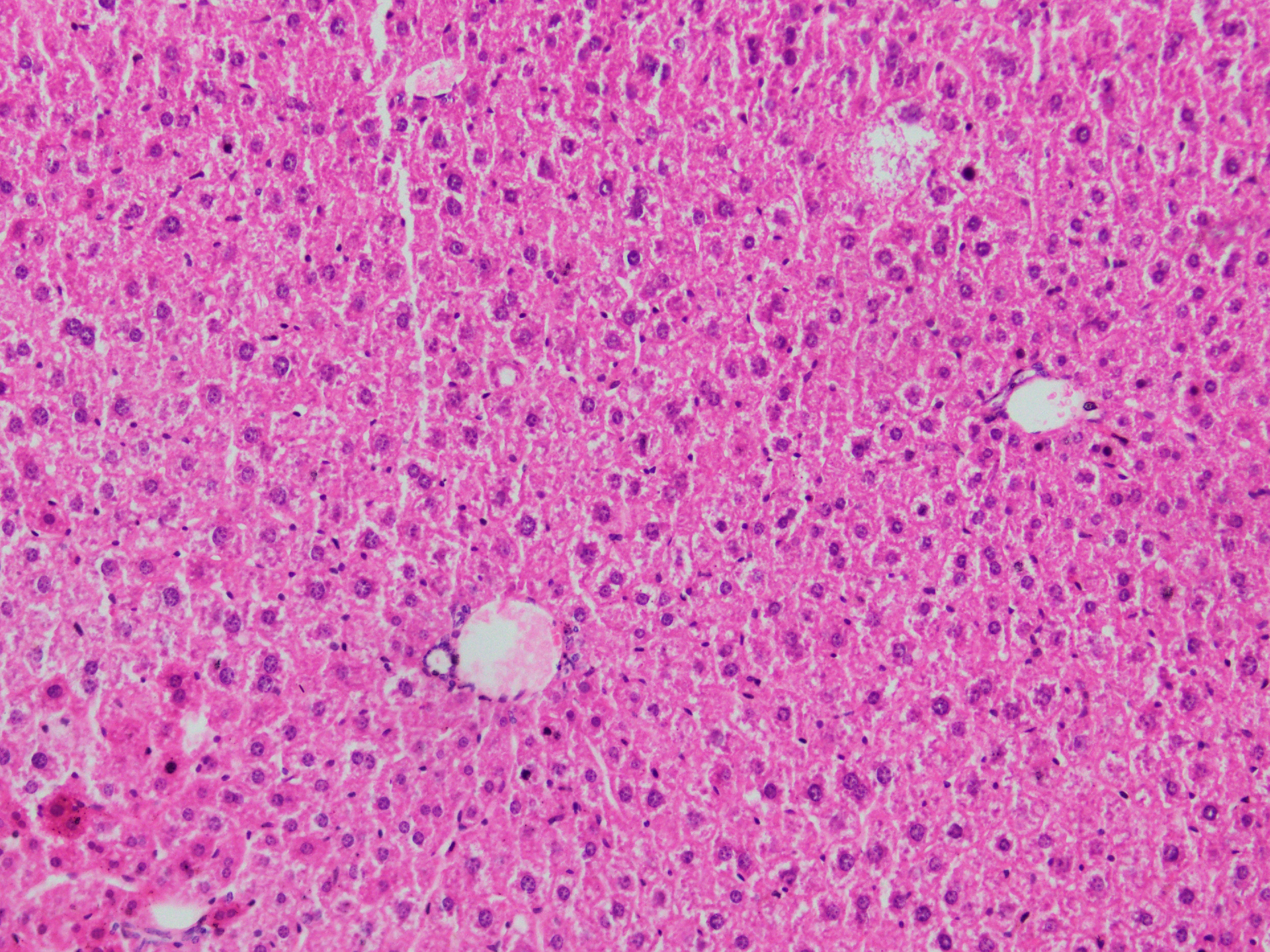

Supplement: Supplementary file 4 [file DataSheet4.ZIP › Liver HE 1/MET.tif]

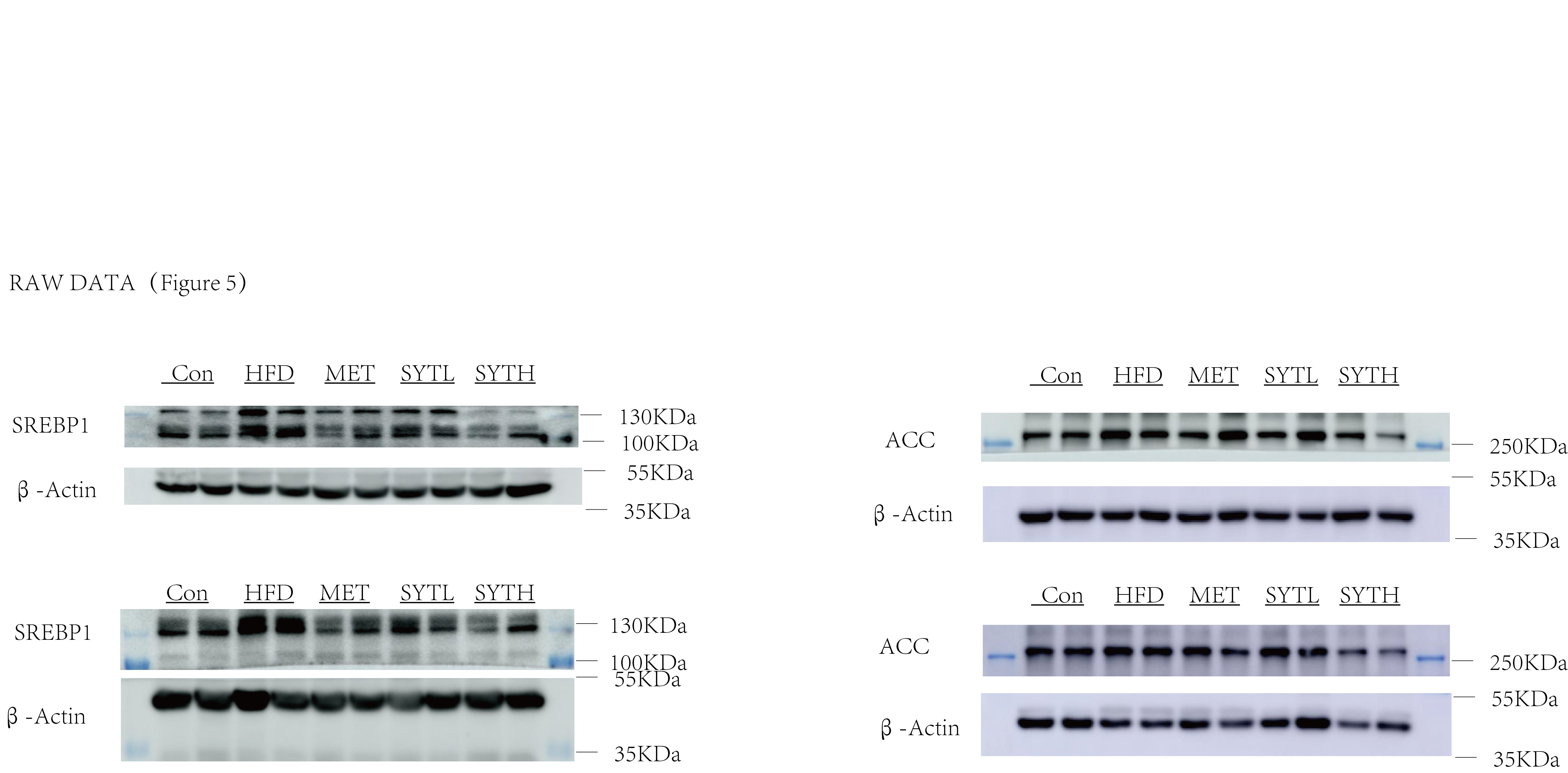

Supplement: Supplementary file 5 [file DataSheet1.ZIP › raw data/raw data figure 6.tif]

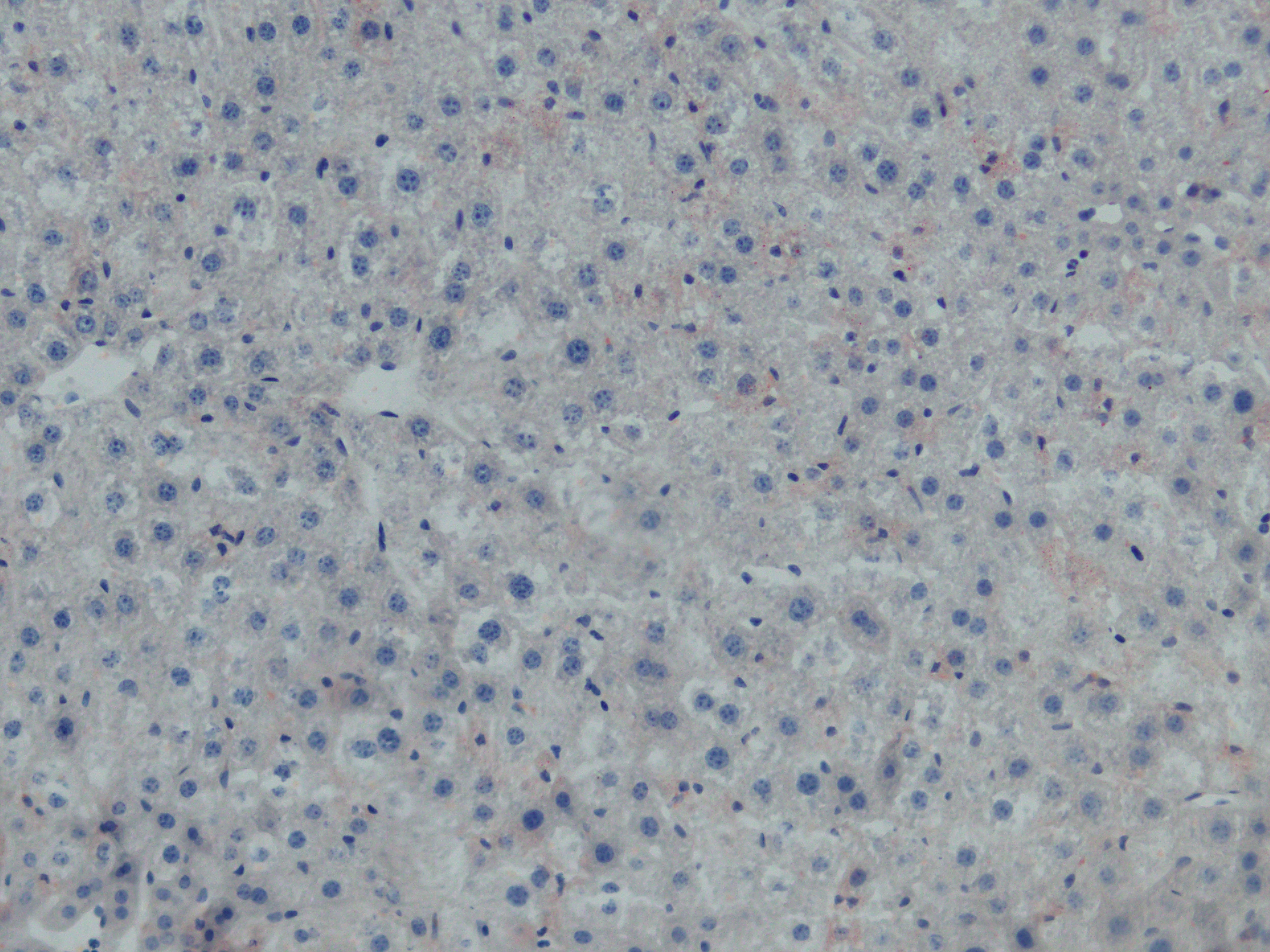

Supplement: Supplementary file 6 [file DataSheet6.ZIP › Liver Oil red O 1/C.tif]

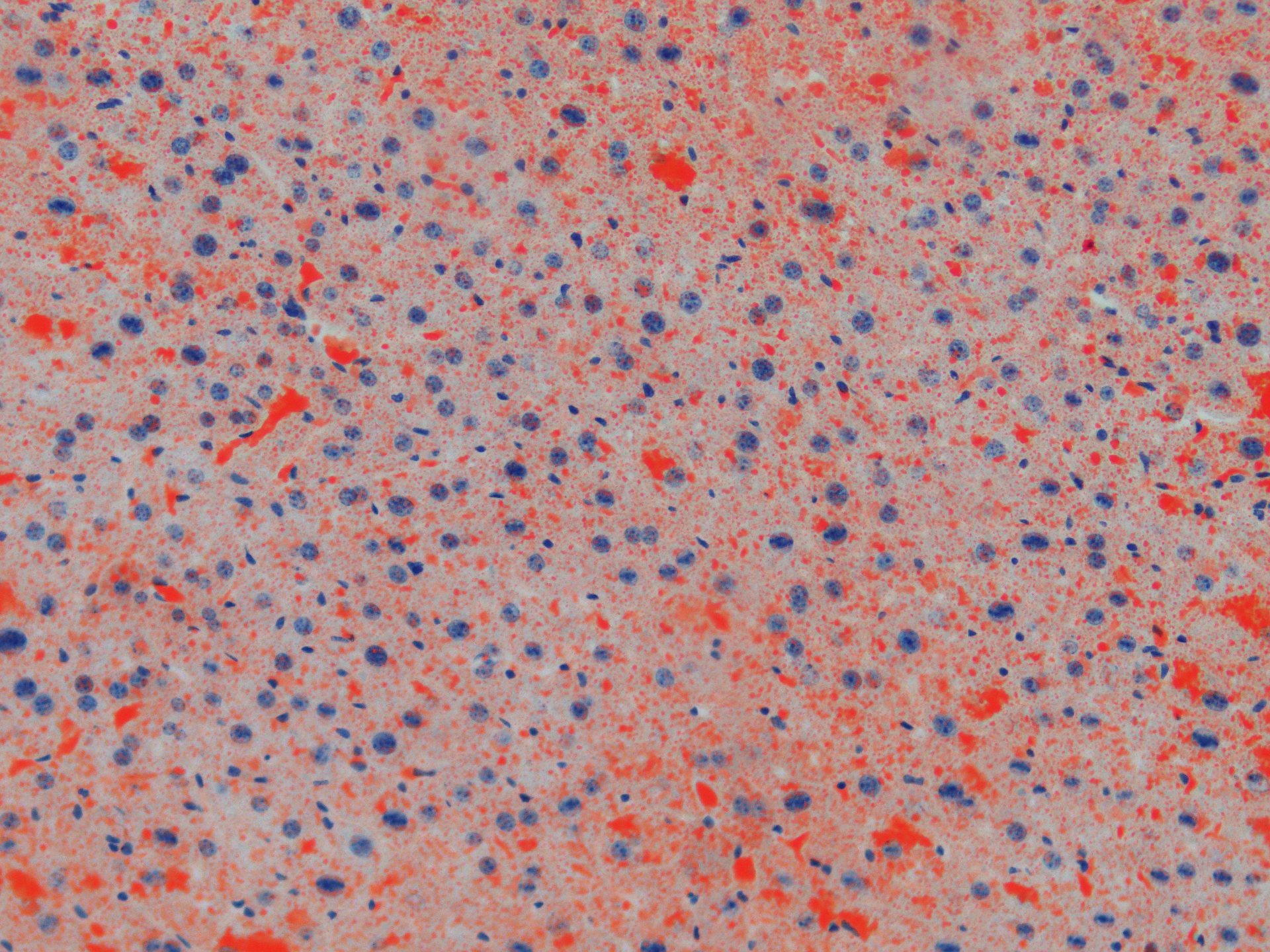

Supplement: Supplementary file 6 [file DataSheet6.ZIP › Liver Oil red O 1/HFD.tif]

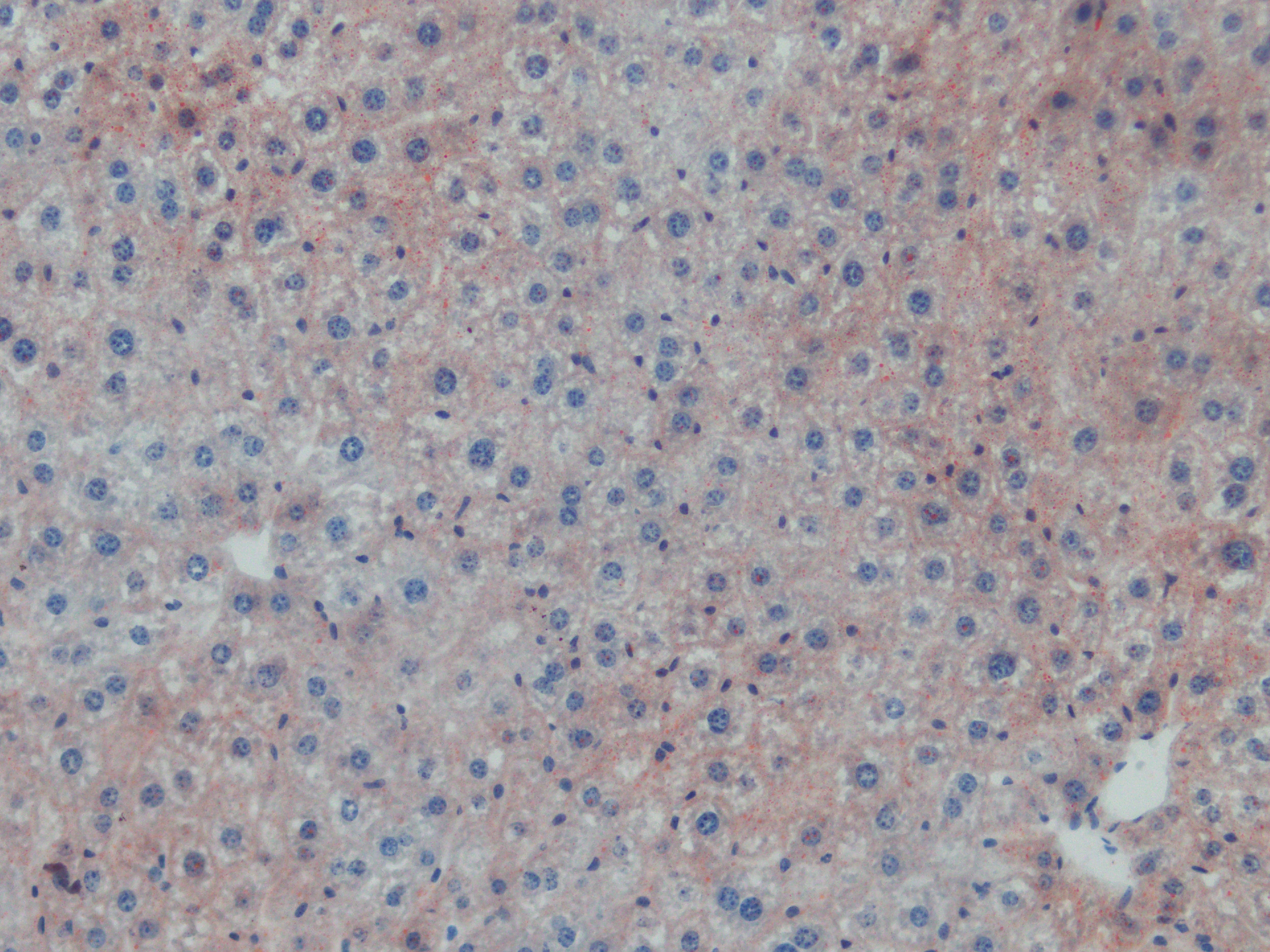

Supplement: Supplementary file 6 [file DataSheet6.ZIP › Liver Oil red O 1/MET.tif]

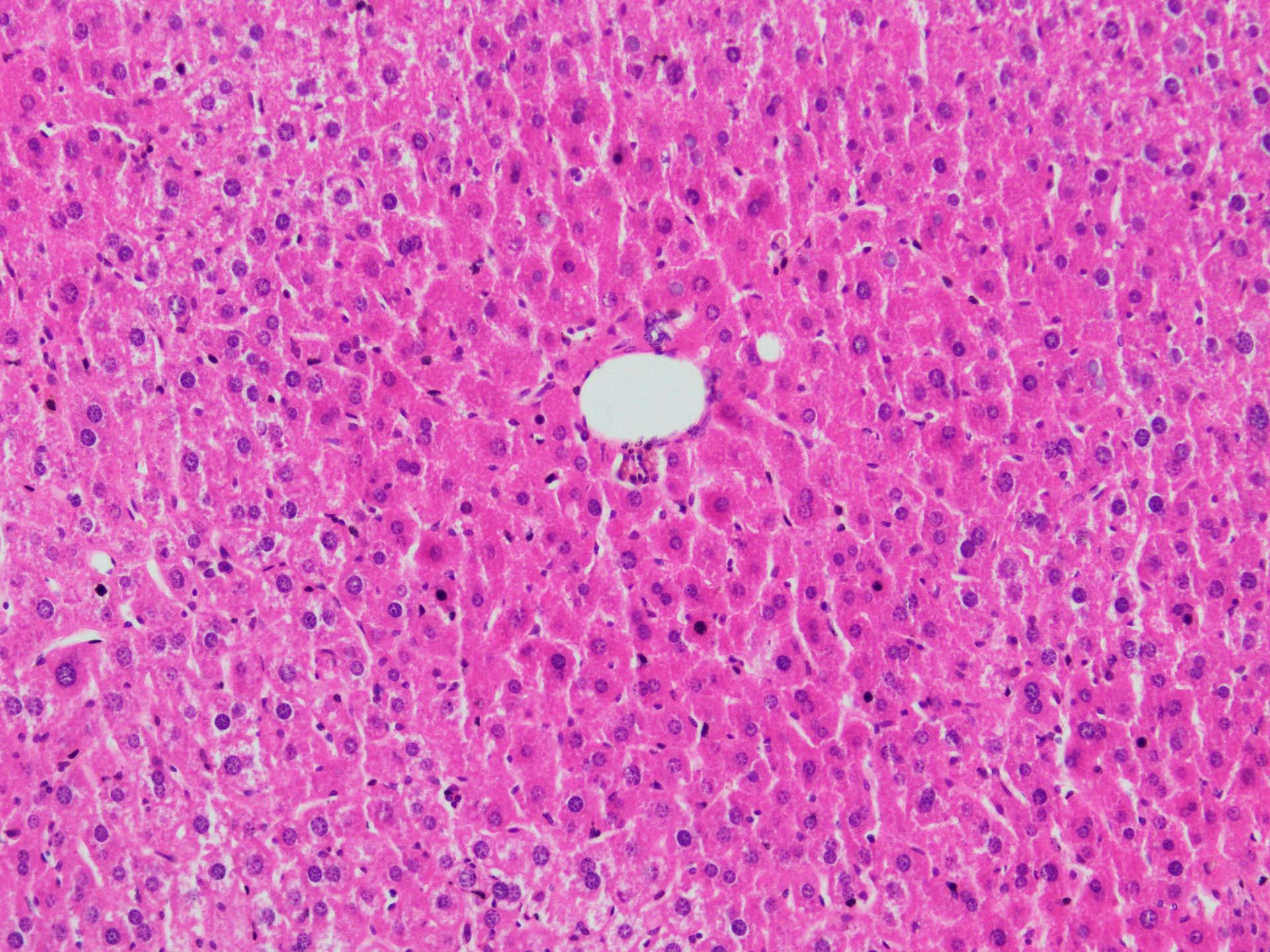

Supplement: Supplementary file 10 [file DataSheet5.ZIP › Liver HE 2/SYTH.tif]

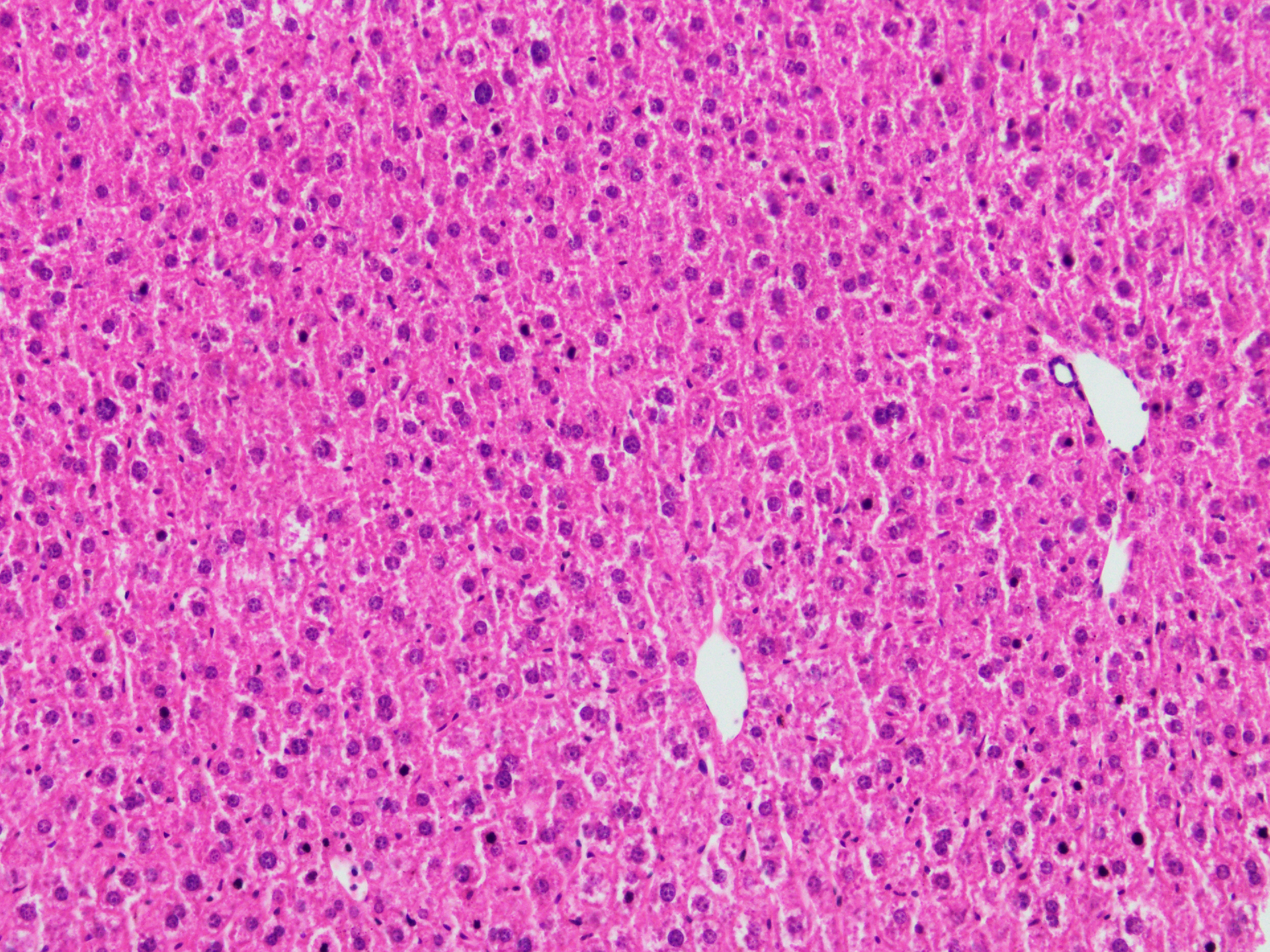

Supplement: Supplementary file 10 [file DataSheet5.ZIP › Liver HE 2/SYTL.tif]

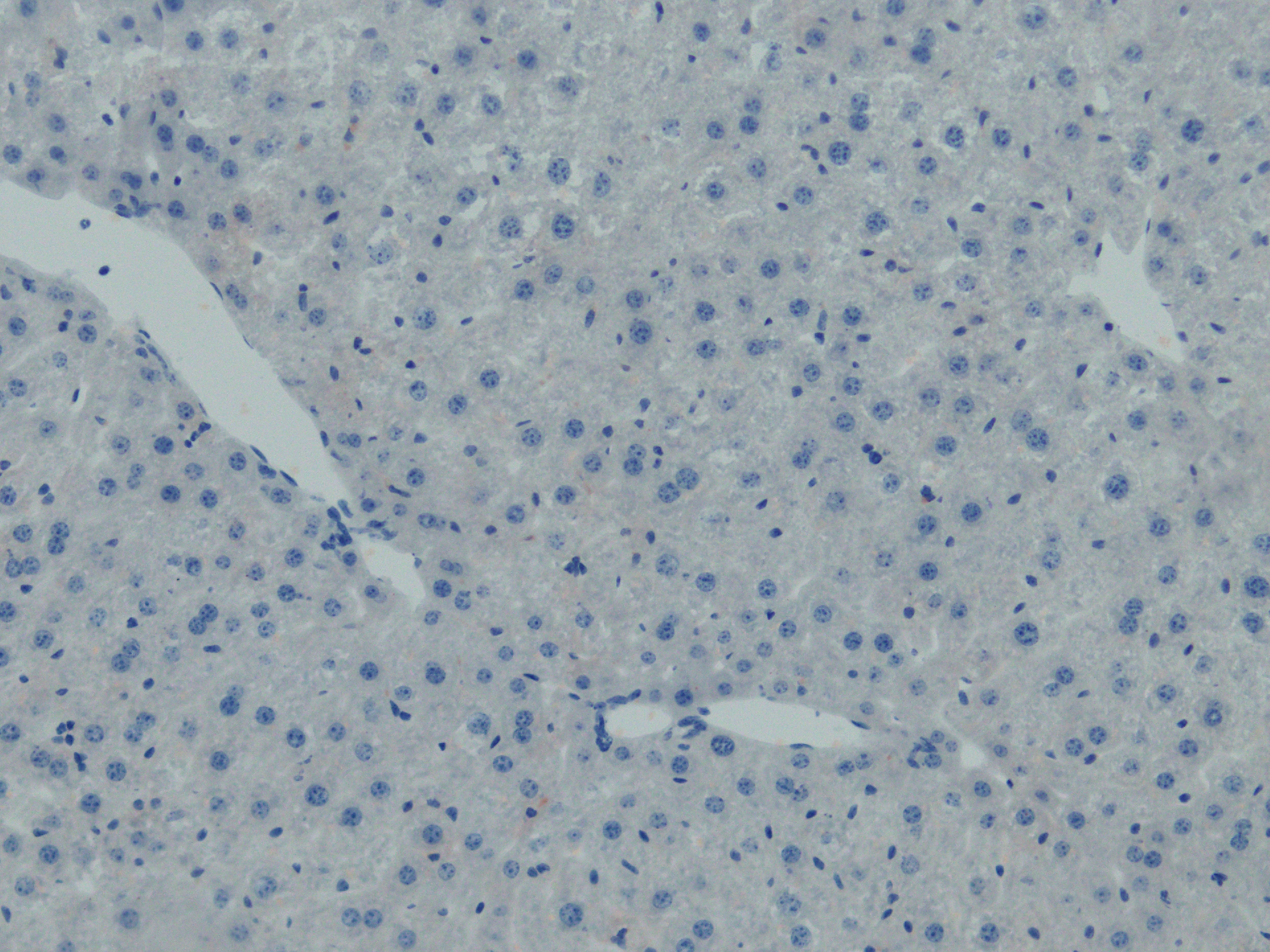

Supplement: Supplementary file 11 [file DataSheet7.ZIP › Liver Oil red O 2/SYTH.tif]

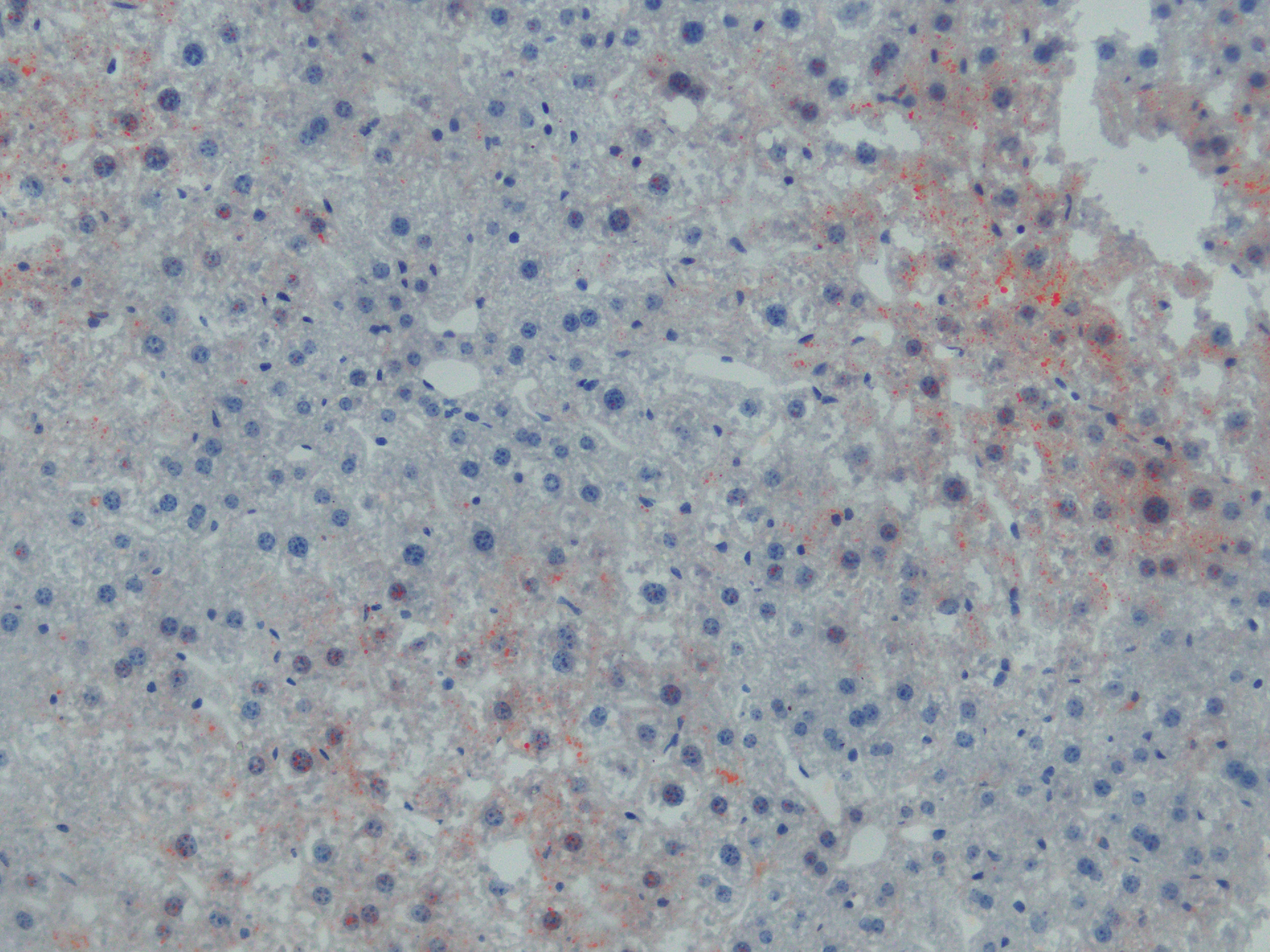

Supplement: Supplementary file 11 [file DataSheet7.ZIP › Liver Oil red O 2/SYTL.tif]
