# Supplementary material for: Sanye Tablet Ameliorates Insulin Resistance and Dysregulated Lipid Metabolism in High-Fat Diet-Induced Obese Mice
Source: Front Pharmacol. 2021 Sep 29;12:713750. doi: 10.3389/fphar.2021.713750 (PMC8511530; doi:10.3389/fphar.2021.713750)
Supplement: Supplementary file 3 [file Presentation1.pdf]

Supplementary methods

Preparation of SYT

SYT was provided by Shandong Buchang Pharmaceuticals Co., Ltd (Shandong, China). In general, the proportion of *Morus alba L.* Leaf, *Nelumbo nucifera Gaertn.* Leaf, *Crataegus pinnatifida Bunge* Leaf, *Salvia miltiorrhiza Bunge* Root, *Paeonia lactiflora Pall.* Root in the SYT was 4:4:4:5:5. The herbs were identified as authentic by Prof. Tianxiang Li (Tianjin University of Traditional Chinese medicine). The *Morus alba L.* Leaf and *Nelumbo nucifera Gaertn.* Leaf were extracted together with 50% ethanol (v/v) under reflux. The decoction was filtered, and then the filtrates were concentrated into dried extract powder. The *Crataegus pinnatifida Bunge* Leaf was extracted with 70% ethanol (v/v) under reflux. Then the extract was adsorbed with macroporous adsorption resin D101, and eluted with 70% ethanol (v/v) to obtained the eluent. After that, the eluent was concentrated into dried extract powder. *Salvia miltiorrhiza Bunge* Root was extracted by percolation with 50% ethanol (v/v). *Paeonia lactiflora Pall.* Root was extracted with distilled water. The absorption and elution processes of *Salvia miltiorrhiza Bunge* Root and *Paeonia lactiflora Pall.* Root were the same as that of *Crataegus pinnatifida Bunge* Leaf. Finally, the eluents were concentrated into dried extract powder, respectively. The yields of the four extracts including *Morus alba L.* Leaf and *Nelumbo nucifera Gaertn.* Leaf, *Crataegus pinnatifida Bunge* Leaf, *Salvia miltiorrhiza Bunge* Root, *Paeonia lactiflora Pall.* Root were 19.75%, 6.25%, 6.3%, and 4.7%, respectively.
