# Supplementary material for: Sanye Tablet Ameliorates Insulin Resistance and Dysregulated Lipid Metabolism in High-Fat Diet-Induced Obese Mice
Source: Front Pharmacol. 2021 Sep 29;12:713750. doi: 10.3389/fphar.2021.713750 (PMC8511530; doi:10.3389/fphar.2021.713750)
Supplement: Supplementary file 7 [file Table2.docx]

Supplementary Material

**Supplementary table S2**

The protein targets of seven well-known compounds from SYT.

| Molecule ID | Molecule Name | Target ID | Target name | 52 common targets related to T2D (marked with *) |
| --- | --- | --- | --- | --- |
| MOL007134 | Danshensu | HBTAR000031 | ACHE | * |
|  |  | HBTAR000043 | ACTB |  |
|  |  | HBTAR000095 | ADRA2A | * |
|  |  | HBTAR000098 | ADRB1 |  |
|  |  | HBTAR000099 | ADRB2 | * |
|  |  | HBTAR000776 | COL1A1 | * |
|  |  | HBTAR000779 | COL3A1 | * |
|  |  | HBTAR001851 | HMOX1 | * |
|  |  | HBTAR002403 | MAOB |  |
|  |  | HBTAR002691 | NF1 |  |
|  |  | HBTAR002755 | NPPA | * |
|  |  | HBTAR003029 | PLAU |  |
|  |  | HBTAR003294 | PTGS1 | * |
|  |  | HBTAR003295 | PTGS2 | * |
|  |  | HBTAR003434 | RELA | * |
|  |  | HBTAR003849 | SOD1 | * |
|  |  | HBTAR004072 | TGFB1 | * |
|  |  | HBTAR004104 | TIMP1 | * |
|  |  | HBTAR000092 | ADRA1D |  |
|  |  | HBTAR002355 | LTA4H |  |
|  |  | HBTAR003223 | PRSS1 |  |
|  |  | HBTAR001094 | DPEP1 |  |
|  |  | HBTAR000096 | ADRA2B | * |
|  |  | HBTAR000460 | CA2 |  |
|  |  | HBTAR000924 | CTRB1 |  |
|  |  | HBTAR002737 | NOS2 | * |
|  |  | HBTAR000634 | CDK2 |  |
|  |  | HBTAR001096 | DPP4 | * |
|  |  | HBTAR000097 | ADRA2C | * |
| MOL003066 | Neochlorogenic acid | HBTAR001094 | DPEP1 |  |
|  |  | HBTAR002737 | NOS2 | * |
|  |  | HBTAR003295 | PTGS2 | * |
|  |  | HBTAR000460 | CA2 |  |
|  |  | HBTAR001096 | DPP4 | * |
|  |  | HBTAR001277 | ESR1 | * |
|  |  | HBTAR000634 | CDK2 |  |
|  |  | HBTAR003223 | PRSS1 |  |
|  |  | HBTAR001710 | GSK3B | * |
|  |  | HBTAR000233 | AR |  |
| MOL001924 | Paeoniflorin | HBTAR000574 | CD14 | * |
|  |  | HBTAR002055 | IL6 | * |
|  |  | HBTAR002291 | LBP | * |
|  |  | HBTAR004140 | TNF | * |
| MOL000561 | Astragalin | HBTAR001710 | GSK3B | * |
|  |  | HBTAR000460 | CA2 |  |
|  |  | HBTAR001307 | F7 | * |
|  |  | HBTAR000634 | CDK2 |  |
|  |  | HBTAR003295 | PTGS2 | * |
|  |  | HBTAR003112 | PPARG | * |
|  |  | HBTAR002737 | NOS2 | * |
|  |  | HBTAR000686 | CHEK1 |  |
|  |  | HBTAR003223 | PRSS1 |  |
|  |  | HBTAR002192 | KCNH2 | * |
|  |  | HBTAR003005 | PIM1 |  |
|  |  | HBTAR000874 | MAPK14 |  |
|  |  | HBTAR005869 | NCOA2 |  |
|  |  | HBTAR001096 | DPP4 | * |
|  |  | HBTAR000233 | AR |  |
|  |  | HBTAR000549 | CCNA2 |  |
|  |  | HBTAR002221 | KDR | * |
|  |  | HBTAR001278 | ESR2 | * |
|  |  | HBTAR001277 | ESR1 | * |
|  |  | HBTAR003294 | PTGS1 | * |
| MOL011865 | rosmarinic acid | HBTAR000233 | AR |  |
|  |  | HBTAR000437 | C3 | * |
|  |  | HBTAR000444 | C5 |  |
|  |  | HBTAR000517 | CASP3 | * |
|  |  | HBTAR000553 | CCND3 |  |
|  |  | HBTAR000566 | CD247 | * |
|  |  | HBTAR000577 | CD80 |  |
|  |  | HBTAR000578 | CD86 | * |
|  |  | HBTAR000641 | CDKN1A |  |
|  |  | HBTAR000756 | CCR3 |  |
|  |  | HBTAR001277 | ESR1 | * |
|  |  | HBTAR001301 | F2 | * |
|  |  | HBTAR002031 | IGHG1 |  |
|  |  | HBTAR002040 | IKBKB | * |
|  |  | HBTAR002045 | IL2 |  |
|  |  | HBTAR002051 | IL4 | * |
|  |  | HBTAR002053 | IL5 |  |
|  |  | HBTAR002090 | IDO1 |  |
|  |  | HBTAR002142 | EIF6 |  |
|  |  | HBTAR002696 | NFATC3 |  |
|  |  | HBTAR003192 | MAPK1 | * |
|  |  | HBTAR003193 | MAPK3 | * |
|  |  | HBTAR003223 | PRSS1 |  |
|  |  | HBTAR003295 | PTGS2 | * |
|  |  | HBTAR003434 | RELA | * |
|  |  | HBTAR003641 | CCL2 | * |
|  |  | HBTAR003642 | CCL3 |  |
|  |  | HBTAR003647 | CCL11 | * |
|  |  | HBTAR003832 | SNCA | * |
|  |  | HBTAR003926 | STAT1 | * |
|  |  | HBTAR004966 | MGAM | * |
|  |  | HBTAR000634 | CDK2 |  |
|  |  | HBTAR001096 | DPP4 | * |
|  |  | HBTAR003112 | PPARG | * |
|  |  | HBTAR000874 | MAPK14 |  |
|  |  | HBTAR000460 | CA2 |  |
| MOL008884 | lithospermic acid | HBTAR004356 | XDH | * |
|  |  | HBTAR003223 | PRSS1 |  |
| MOL007074 | salvianolic acid B | HBTAR000042 | ACTA2 |  |
|  |  | HBTAR000517 | CASP3 | * |
|  |  | HBTAR000776 | COL1A1 | * |
|  |  | HBTAR001976 | ICAM1 | * |
|  |  | HBTAR002221 | KDR | * |
|  |  | HBTAR002508 | MMP2 | * |
|  |  | HBTAR002850 | SERPINE1 | * |
|  |  | HBTAR002861 | PAWR |  |
|  |  | HBTAR003028 | PLAT | * |
|  |  | HBTAR003178 | PRKCB | * |
|  |  | HBTAR003192 | MAPK1 | * |
|  |  | HBTAR003295 | PTGS2 | * |
|  |  | HBTAR003434 | RELA | * |
|  |  | HBTAR003588 | RYR2 |  |
|  |  | HBTAR004087 | THBD | * |
|  |  | HBTAR004304 | VCAM1 | * |
|  |  | HBTAR004310 | VEGFA | * |
|  |  | HBTAR007419 | DUOX2 |  |
|  |  | HBTAR010124 | SPZ1 |  |
