# Supplementary material for: Sanye Tablet Ameliorates Insulin Resistance and Dysregulated Lipid Metabolism in High-Fat Diet-Induced Obese Mice
Source: Front Pharmacol. 2021 Sep 29;12:713750. doi: 10.3389/fphar.2021.713750 (PMC8511530; doi:10.3389/fphar.2021.713750)
Supplement: Supplementary file 8 [file DataSheet2.ZIP › supplementary data/supplementary data-figure.docx]

Supplementary Material

# Supplementary Figures

## **
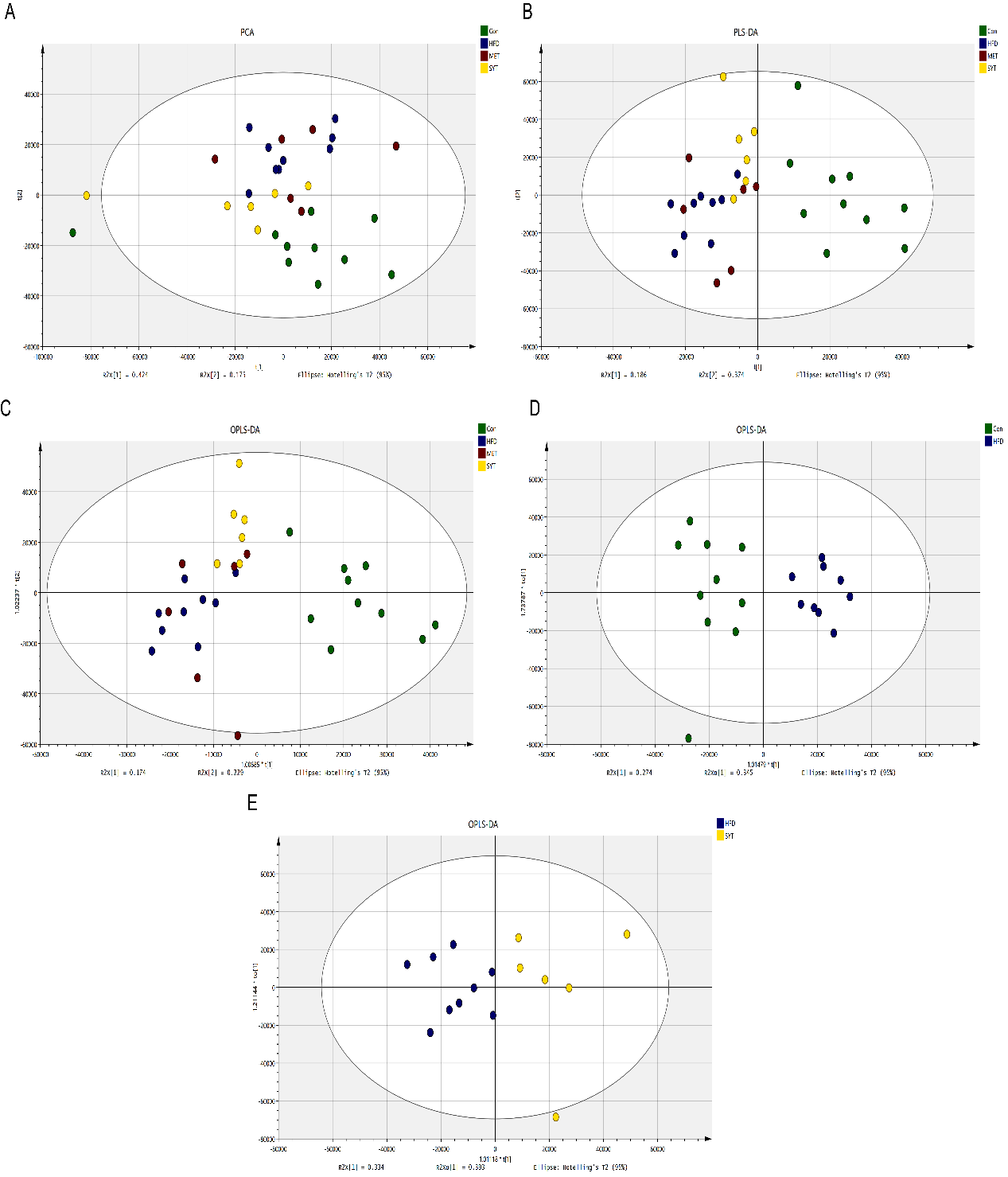
**

**Supplementary Figure 1.** **SYT improves serum lipid metabolism in HFD mice by lipidomics analysis based on UHPLC-MS.** Principal component analysis (PCA) score plot of serum lipid profiling among all groups (A). PLS-DA score plot analysis of serum lipids among all groups (B). OPLS-DA score plot analysis of serum lipids among four groups (C). OPLS-DA plot comparing the HFD and Con groups from serum samples (D). OPLS-DA plot comparing the HFD and SYT groups from serum samples (E).


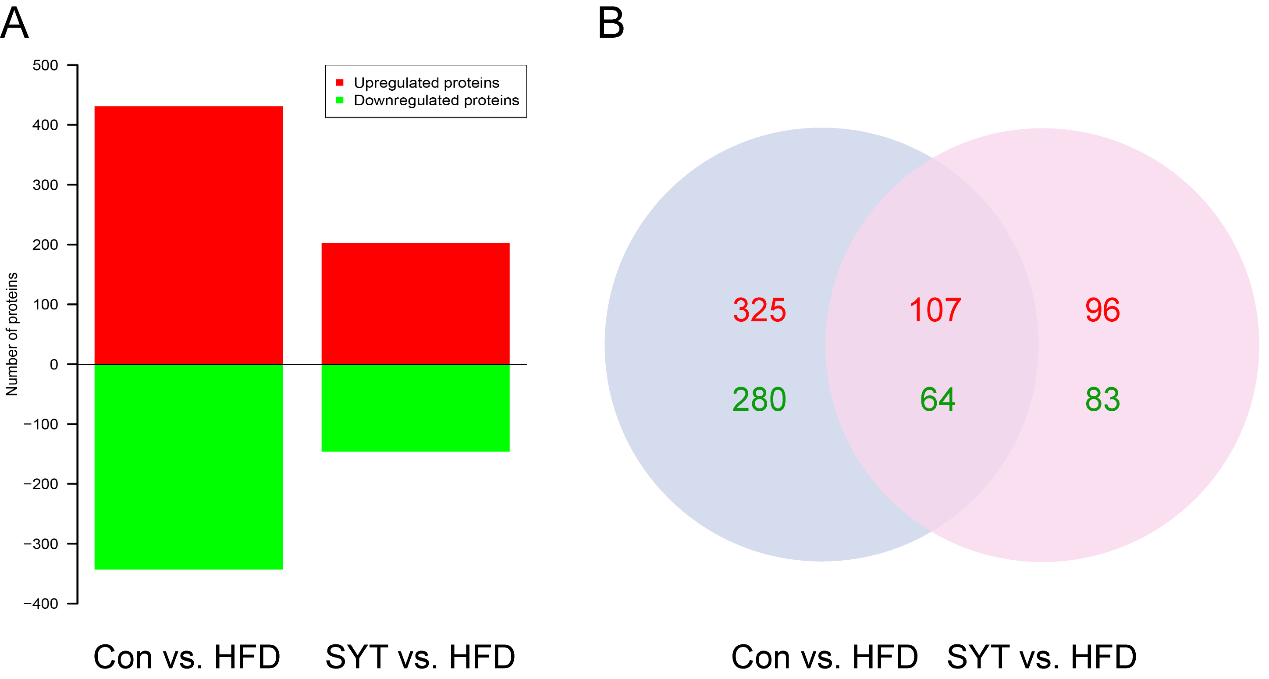


**Supplementary Figure 2.** **Differential proteins.** A. Differential proteins in each comparison group. B. Venn diagram of the distribution and overlaps of differential proteins in each comparison group.


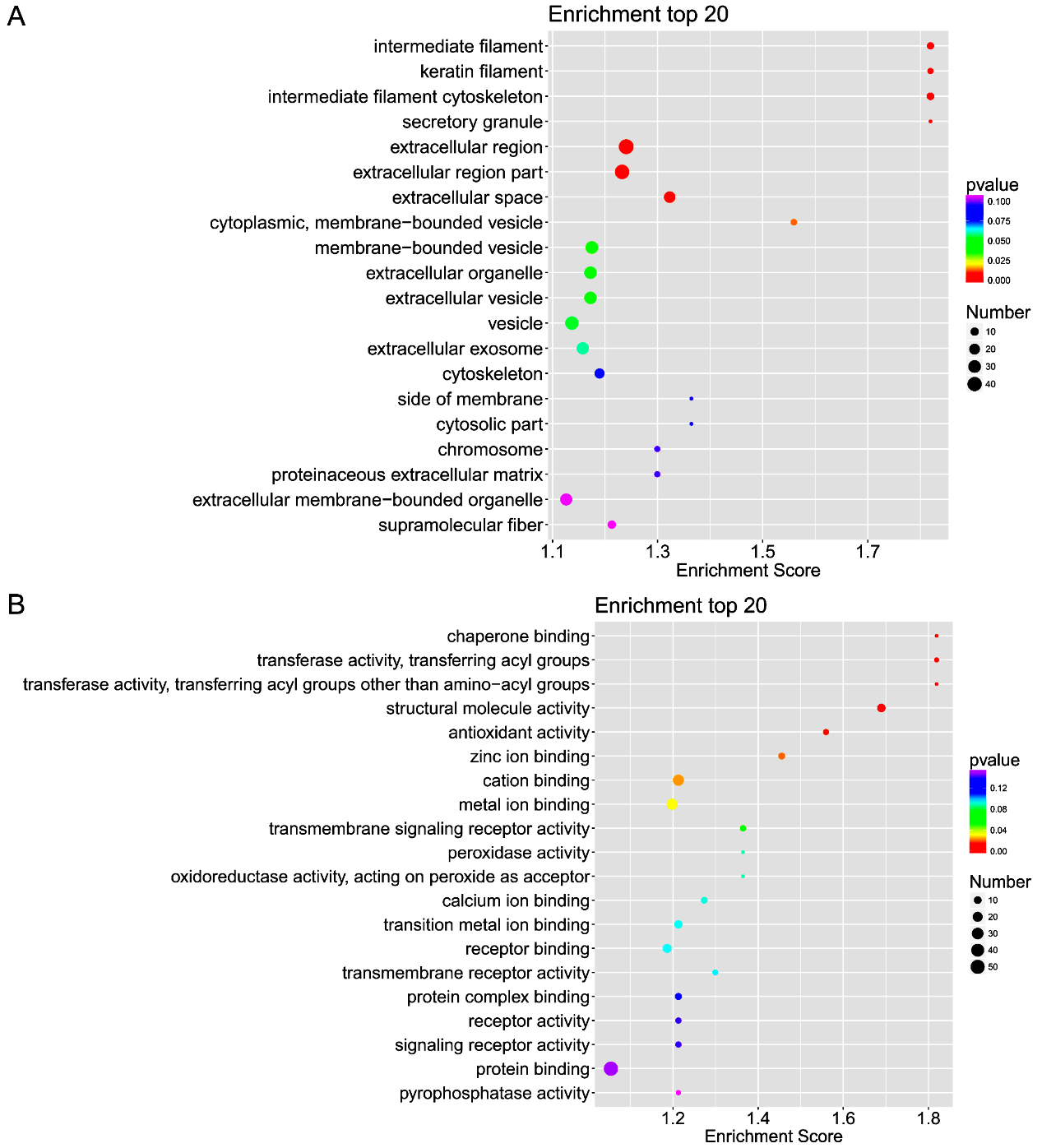


**Supplementary Figure 3. Gene ontology (GO) enrichment analysis.** A. Top 20 cellular component enrichment terms. B. Top 20 MF enrichment terms.


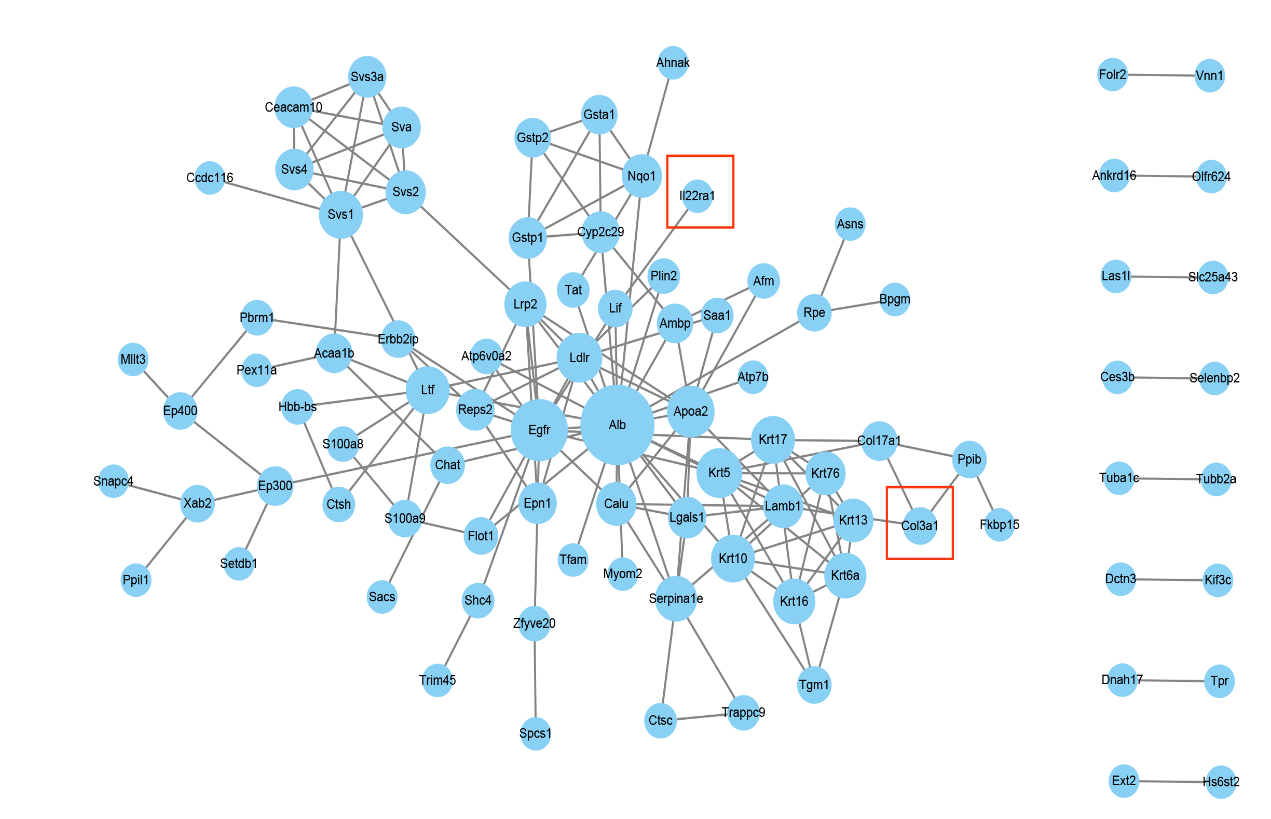


**Supplementary Figure 4. PPI analysis.** Il22ra1 and Col3a1 are labeled by a red rectangle and consistent with GO and KEGG pathway analysis.
